# Hierarchical Clustering of Breast Cancer Methylomes Revealed Differentially Methylated and Expressed Breast Cancer Genes

**DOI:** 10.1371/journal.pone.0118453

**Published:** 2015-02-23

**Authors:** I-Hsuan Lin, Dow-Tien Chen, Yi-Feng Chang, Yu-Ling Lee, Chia-Hsin Su, Ching Cheng, Yi-Chien Tsai, Swee-Chuan Ng, Hsiao-Tan Chen, Mei-Chen Lee, Hong-Wei Chen, Shih-Hui Suen, Yu-Cheng Chen, Tze-Tze Liu, Chuan-Hsiung Chang, Ming-Ta Hsu

**Affiliations:** 1 VGH-YM Genome Center, National Yang-Ming University, Taipei, Taiwan; 2 Institute of Biochemistry and Molecular Biology, National Yang-Ming University, Taipei, Taiwan; 3 Center for Systems and Synthetic Biology, National Yang-Ming University, Taipei, Taiwan; 4 Institute of Biomedical Informatics, National Yang-Ming University, Taipei, Taiwan; University of Bonn, Institut of experimental hematology and transfusion medicine, GERMANY

## Abstract

Oncogenic transformation of normal cells often involves epigenetic alterations, including histone modification and DNA methylation. We conducted whole-genome bisulfite sequencing to determine the DNA methylomes of normal breast, fibroadenoma, invasive ductal carcinomas and MCF7. The emergence, disappearance, expansion and contraction of kilobase-sized hypomethylated regions (HMRs) and the hypomethylation of the megabase-sized partially methylated domains (PMDs) are the major forms of methylation changes observed in breast tumor samples. Hierarchical clustering of HMR revealed tumor-specific hypermethylated clusters and differential methylated enhancers specific to normal or breast cancer cell lines. Joint analysis of gene expression and DNA methylation data of normal breast and breast cancer cells identified differentially methylated and expressed genes associated with breast and/or ovarian cancers in cancer-specific HMR clusters. Furthermore, aberrant patterns of X-chromosome inactivation (XCI) was found in breast cancer cell lines as well as breast tumor samples in the TCGA BRCA (breast invasive carcinoma) dataset. They were characterized with differentially hypermethylated *XIST* promoter, reduced expression of *XIST*, and over-expression of hypomethylated X-linked genes. High expressions of these genes were significantly associated with lower survival rates in breast cancer patients. Comprehensive analysis of the normal and breast tumor methylomes suggests selective targeting of DNA methylation changes during breast cancer progression. The weak causal relationship between DNA methylation and gene expression observed in this study is evident of more complex role of DNA methylation in the regulation of gene expression in human epigenetics that deserves further investigation.

## Introduction

Breast cancer is the most common cancer in women in the world. Since 2008, breast cancer incidence has increased by more than 20% and mortality increased by 14%. Apart from the genetic and hormonal risk factors that predisposing women to breast cancer, other factors such as life-styles, environmental and nutritional also seemed to play a part in this complex, multifactorial disease. Like many cancers, epigenetic dysregulation has been implicated to play a role in breast cancer development [1–3].

DNA methylation is one of the major epigenetic regulatory mechanisms in higher organisms. It plays significant roles in many biological processes, including genomic imprinting, embryonic development, X-chromosome inactivation (XCI), genome stability, suppression of repetitive sequences and tumorigenesis [4–8] DNA methylation involves the addition of a methyl group to the carbon-5 position of cytosine residues at CpG dinucleotides by the DNA methyltransferase enzymes. Of the 28 million CpG sites in the human genome, 70 to 80% are methylated in most cell types [9]. The CpG sites are unevenly distributed in the genome whereby clusters of CpG sites, termed CpG islands (CGIs), are often found at the promoter regions. The regulation of gene expression through differential methylation of the CpG sites within promoter CGIs has been extensively studied [10–12]. Promoter CGIs are often found unmethylated and this state is associated with gene activation whereas gene silencing is often associated with promoter CGIs methylation. DNA methylation is a relatively stable epigenetic trait, hence aberrant promoter (de-)methylation often leads to adverse alteration in gene expression, and such event is one of the major hallmarks of tumor progression [13–17]. DNA methylation changes may occur at regions immediately adjacent to CGIs (CGI shores), and at CpG sites far away from CGIs and/or promoters in cancer cells [18–22]. The term “hypomethylated region” (HMR) was used to describe small genomic loci (usually less than 50 Kb) that were lowly methylated or unmethylated. HMRs found at non-promoter regions may mark cryptic promoters and enhancers associated with tissue-specificity [23–25].

There are currently four genome-wide methylation profiling technologies to study DNA methylation in a high-throughput manner, namely whole-genome bisulfite sequencing (WGBS), enrichment-based sequencing, reduced representation bisulfite sequencing and the Infinium HumanMethylation BeadChip which is a low-cost alternative to the sequencing-based methods [26]. Past array-based DNA methylation studies have concluded that the unusual hypermethylation of a number of CpG loci were receptor specific in breast tumors [27–30]. Aberrant hypermethylation of certain genes have been significantly associated with worse outcome, and some were associated with increased risk of developing metastases [28, 29, 31]. While at a higher cost, the WGBS provides the whole-genome coverage at a single-nucleotide resolution and is considered the gold-standard approach for quantitative measurement of methylation level. Since 2009, several human WGBS studies have been conducted to explore the DNA methylation landscapes in various tissue types and cell lines, at different age, as well as between normal and diseased states [32–41]. With regards to the breast cancer research, Hon *et al* decoded the methylomes of HMEC and HCC1954, cell lines derived from breast epithelium and breast carcinoma respectively [38]. In this work, the authors confirmed the presence of extensive DNA hypomethylation at the partially methylated domains (PMDs), a term used to describe large genomic blocks with abnormal hypomethylation observed in cancer methylomes and extra-embryonic tissues, in HCC1954. The global DNA hypomethylation was associated with several compensatory repressive mechanisms.

In this study, we performed WGBS on a normal human breast tissue, a benign fibroadenoma, two invasive breast carcinomas and breast adenocarcinoma cell line MCF7 to investigate the DNA methylation changes in normal and cancerous breast cells. In addition, chromatin immunoprecipitation (ChIP) and transcriptome analysis was performed to investigate the relationship between differential methylation and differential gene expression and also with gene regulation potential. We identified two main forms of DNA methylation changes in breast tumor samples: (1) the differential methylation of the kilobase-sized HMRs and (2) the hypomethylation of the megabase-sized PMDs. Hierarchical clustering of HMRs revealed specific groups of genes and enhancer sites differentially methylated in breast cancer. The analysis also showed aberrant XCI in breast cancer cell lines and in almost half of the primary tumor samples in TCGA breast invasive carcinoma (BRCA) dataset. The disruption of XCI impacts gene regulation epigenetically and transcriptionally, as well as breast cancer survival.

## Results

### CpG density-related DNA methylation variations in breast cancer methylomes

We carried out WGBS experiments on a normal human breast sample (NB), a fibroadenoma (BT089), two invasive ductal carcinomas (BT126 and BT198) and the breast adenocarcinoma cell line MCF7. We generated an average of 405 million pairs of reads per sample, whereby 322 million pairs (79%) were aligned to the hg18 reference genome, resulting in an average sequencing depth of 18.8-fold. An average of 26 million (91.3%) CpG sites were covered and the bisulfite conversion rate was determined to be at least 99% based on the alignment with *in silico* converted non-CpG cytosines. We included the published methylation data of a normal (HMEC, derived from breast epithelium) and a breast cancer cell line (HCC1954, derived from ductal breast carcinoma) for comparative analysis [38]. The statistics of all seven WGBS were summarized in S1 Table.

**Fig 1:**
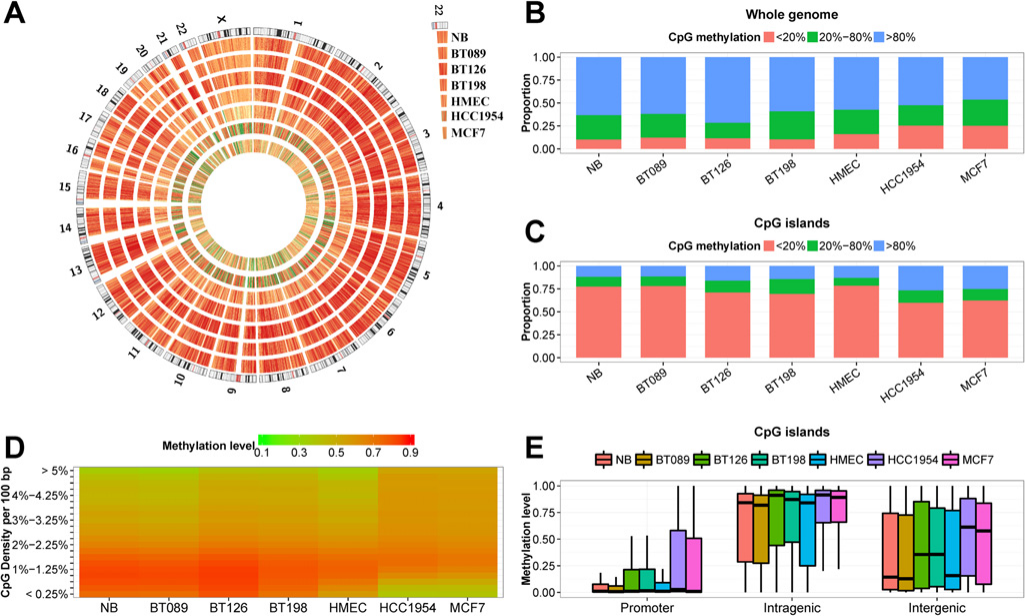
WGBS of a normal breast (NB), three primary breast tumors (BT089, BT126 and BT198), a mammary epithelial cell line (HMEC) and two breast cancer cell lines (MCF7 an HCC1954). (A) Circos representation of genome-wide DNA methylation levels in the seven breast samples. The data represent the average methylation levels for all of the CpGs in 56,779 50 Kb windows. Coloring indicates methylation levels from low (green) to high (red). (B) The proportion of CpG sites in the DNA that were lowly (< 20%), intermediately (20% ~ 80%) and highly (> 80%) methylated in the seven samples. (C) The proportion of CpG sites in the CGI that were lowly (< 20%), intermediately (20% ~ 80%) and highly (> 80%) methylated in the seven samples. (D) Heatmap representation of average methylation levels of 10 Kb windows with different CpG densities. The CpG density was expressed as the number of CpG sites per 100 bp of nucleotide sequence. Coloring indicates methylation levels from low (green) to high (red). (E) The distribution of the DNA methylation levels of CGI in promoter (TSS ± 1 Kb), intragenic and intergenic regions.

We showed in S1 Table that the mean DNA methylation levels of the breast cell lines (HMEC, HCC1954 and MCF7) were lower than the normal and primary tumor samples (t-test p-value = 0.0199). Graphically, Fig. 1A and Fig. 1B showed the cell lines were more lowly methylated in a genome-wide manner. In CGIs, there were fewer lowly methylated CpG sites in HCC1954, MCF7 and the two invasive carcinomas (BT126 and BT198) than normal breast (t-test p-value = 0.0196; Fig. 1C). We divided the hg18 reference genome into 10,000-bp bins and calculated the methylation levels and CpG density at each bin for all seven methylomes. Fig. 1D showed that the CpG-rich regions were more hypermethylated in BT126, BT198, HCC1954 and MCF7, whereas the CpG-poor regions were hypomethylated in the cell lines.

We then examined the methylation levels of CGIs at various genomic locations (Fig. 1E). More than 80% of the promoter CGIs in NB, HMEC, and fibroadenoma BT089 remain lowly methylated (< 20% methylation), whereas only 70% were lowly methylated in primary tumors and tumor cell lines. The non-promoter CGIs had varying degrees of DNA methylation in all samples, but were more methylated in tumor samples. These observations are consistent with previous reports of aberrant hypermethylation of CGIs in cancers [42].

### Contraction and expansion of hypomethylated regions (HMRs) in breast tumors

We identified between 53,000 and 116,000 hypomethylated regions (HMRs) in the seven breast methylomes. The breast cancer cell lines (MCF7 and HCC1954) had more and wider HMRs than the other breast methylomes, and NB had the least number of HMRs (S2 Table). The additional HMRs identified in tumors and cell lines tend to occur at regions of lower CpG densities. The HMRs in NB are 1.5 to 2.5 times wider than the CGI that they intersected with, and the hypomethylated CGI shore regions were enriched with regulatory elements (S1A and B Fig.). We compared the HMRs identified in NB with those in the other six breast methylomes to examine the expansion and contraction of HMRs (S1C Fig.). More than 45% of the NB HMRs showed little change in width in fibroadenoma (BT089), whereas close to 50% of NB HMRs became widened in the two invasive carcinomas (BT126 and BT198) and the three cell lines. Extreme widening (more than eight time the widths in NB) of 14% of NB HMRs were observed in both MCF7 and HCC1954. In generally, non-CGI associated NB HMRs tend to become expanded in tumor cells, whereas the CGI-containing counterparts underwent contraction. This is in agreement with the aberrant hypermethylation of CGIs and CpG-rich regions in tumor cells showed in Fig. 1.

Although NB and the benign breast tumor BT089 have similar DNA methylation patterns, about 40% of NB HMRs were slightly contracted or expanded in BT089. Interestingly, among the 4,409 expanded or contracted promoter HMRs in BT089 we identified 27 tumor suppressor genes (TSGs) and 304 transcription regulators (S3 Table). We analyzed the “Stage 1” paired tumor-normal RNA expression of the breast invasive carcinoma (BRCA) dataset from TCGA. Of the 27 TSGs, twelve with HMR contraction and six with HMR expansion in BT089 were found respectively under-expressed and over-expressed in BRCA tumor samples (FDR ≤ 0.05). As for transcription regulators, 29 of 71 under-expressed genes had contracted HMRs and 28 of 50 over-expressed genes had expanded HMRs in BT089. This result suggests that there are tumor-specific epigenetic changes in genes encoding for transcription regulators and tumor suppressors in benign and early stage breast tumors.

### Hierarchical clustering analysis of hypomethylated regions (HMRs) revealed tumor-specific HMRs

**Fig 2:**
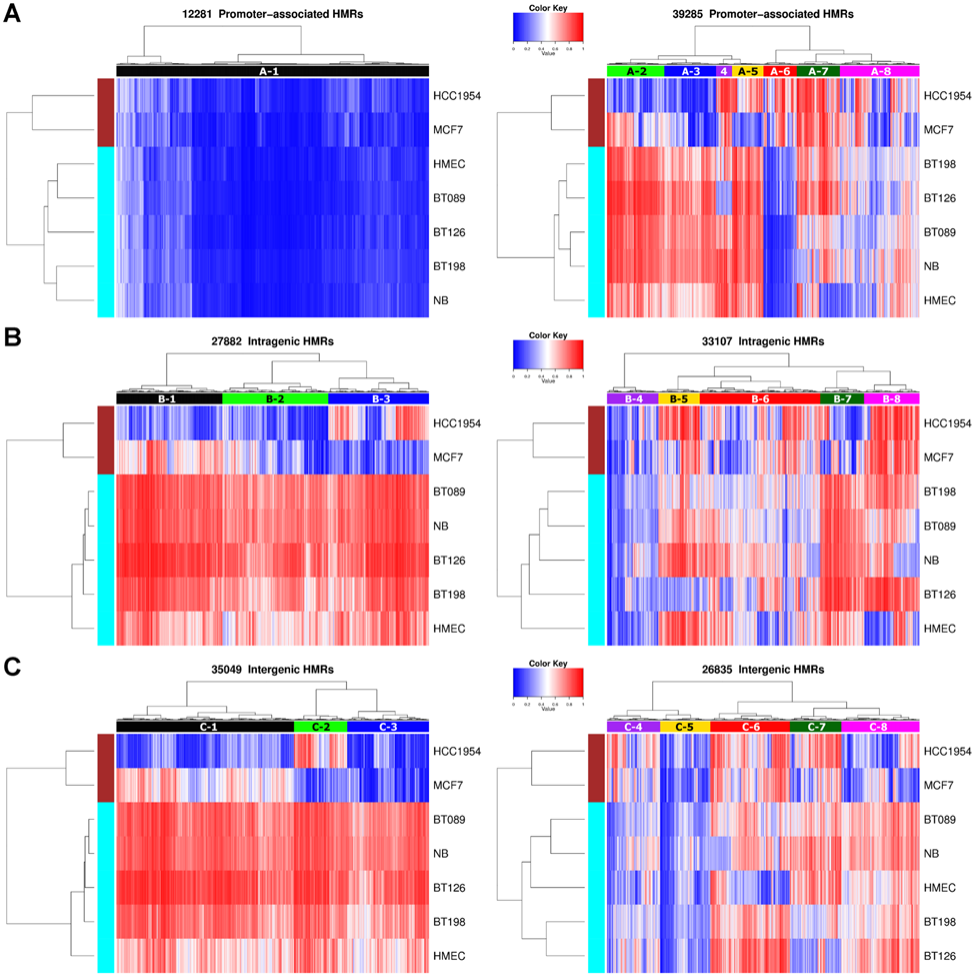
Hierarchical clustering of promoter, intragenic and intergenic HMRs according to their DNA methylation levels in the seven breast methylomes. Coloring indicates methylation levels from low (blue) to high (red). Eight distinctive HMR clusters were indicated in each of the three genomic locations. (A) Promoter HMRs. The A-1 cluster represents the promoter HMRs that were consistently lowly methylated in all seven samples. (B) Intragenic HMRs. The B-1, B-2 and B-3 clusters were hypomethylated in one or both cancer cell lines (MCF7 and HCC1954), while other samples remain methylated. (C) Intergenic HMRs. The C-1, C-2 and C-3 clusters were hypomethylated in one or both cancer cell lines (MCF7 and HCC1954), while other samples remain methylated.

To discover tumor-specific differential methylation at HMRs, we merged HMRs identified in the seven methylomes to create a reference set of HMRs, and performed hierarchical clustering based on their methylation profiles (Fig. 2). This generated eight promoter (A-type), eight intragenic (B-type) and eight intergenic (C-type) clusters. A large proportion of the promoter HMRs, forming the A-1 cluster, were lowly methylated across all samples. Other A-type clusters showed sample-specific DNA methylation patterns. For example, the A-6 HMRs were hypermethylated only in MCF7 and HCC1954, whereas A-7 HMRs were hypermethylated in MCF7 and HCC1954 as well as in primary tumors. Clustering analysis also revealed cancer cell line-specific intragenic and intergenic HMRs, such as B-1 and C-1 HMRs, where they remained methylated in normal and primary tumors.

### Negative correlation between differential methylation and gene expression of X-linked A-8 associated breast cancer genes

**Fig 3:**
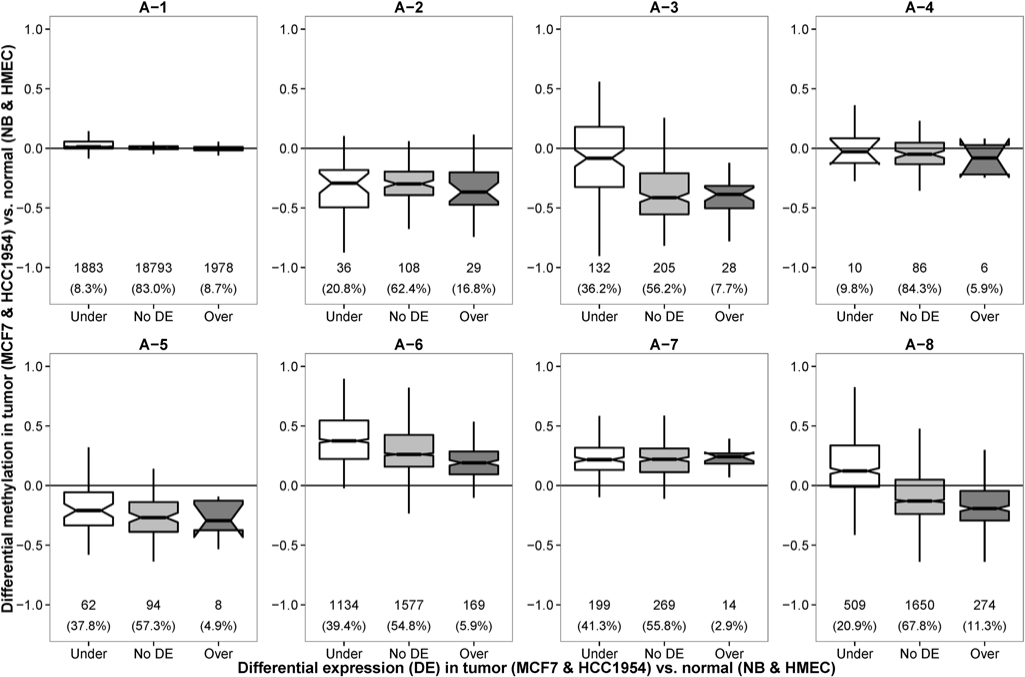
Distribution of differential methylation levels of gene promoters for under-expressed, over-expressed and not differentially expressed genes between breast cancer cell lines (MCF7 and HCC1954) and normal breast samples (NB and HMEC). The differential methylation and differential gene expression between breast cancer cell lines and normal samples were negative correlated in A-3, A-6 and A-8 promoter HMRs. In other HMR clusters, the distributions of differential methylation levels among under-expressed (Under), over-expressed (Over) and not differentially expressed (No DE) genes were similar in form.

There were little methylation changes in A-1 and A-4 HMRs between normal (NB and HMEC) and tumor (MCF7 and HCC1954) samples (t-test p-value = 0.0835). In addition, more than 80% of the transcripts associated with these HMRs had no differential expressions (Fig. 3). Functional analysis using Ingenuity pathway analysis (IPA) showed these genes participate in housekeeping functions, such as transcription, protein metabolism and cellular assembly and organization (S4 Table).

Negative correlation between methylation and expression was observed in A-8 promoter HMRs. Functional analysis of differentially expressed genes associated with A-8 HMRs showed enrichment of genes located on the X chromosome (p-value = 8.47E-14) and breast and/or ovarian cancer (p-value = 1.71E-09) (S5 Table). Interestingly, although the A-3 and A-6 HMRs are differentially hypomethylated and hypermethylated in tumors respectively, the extent of hypomethylation and hypermethylation seemed to exert a negative effect on the RNA expression (Fig. 3). In both cases, the under-expressed genes exhibited highest degree of differential methylation and the over-expressed genes had lowest differential methylation. IPA showed differentially expressed genes that have A-3 promoters are associated with melanoma (p-value = 1.49E-07), whereas those with A-6 promoters are enriched with breast and/or ovarian cancer (p-value = 1.07E-12) (S5 Table). As for A-2, A-5 and A-7 promoter HMRs, the under-, over- and not differentially expressed genes had similar differential methylation patterns, suggesting that promoter methylation does not play key role in the transcriptional regulation of these genes. Many of the differentially expressed genes from these three clusters were also cancer-associated as revealed by IPA (S6 Table). We verified three genes that exhibit negative correlation between promoter methylation and genes expression (S2 Fig.).

### Promoter hypermethylation of A-6 associated breast cancer genes in clinical tumor tissues

We analyzed the TCGA BRCA methylation dataset to verify the methylation status of HMRs in this larger and independent cohort. The methylation profiles of 98 normal and 743 tumor samples were generated using the Infinium HumanMethylation450 BeadChip. The microarray assays 485,577 CpG sites, which covers about 1.7% of total CpG sites in the human genome. Almost 60% of the CpG sites interrogated by the microarray were located near TSS. However, more than 70% of HMRs identified from our WGBS assays were located in the intragenic or intergenic regions. As a result, only 17,502 HMRs that have sufficient CpG coverage in the microarray were analyzed here (S7 Table).

**Fig 4:**
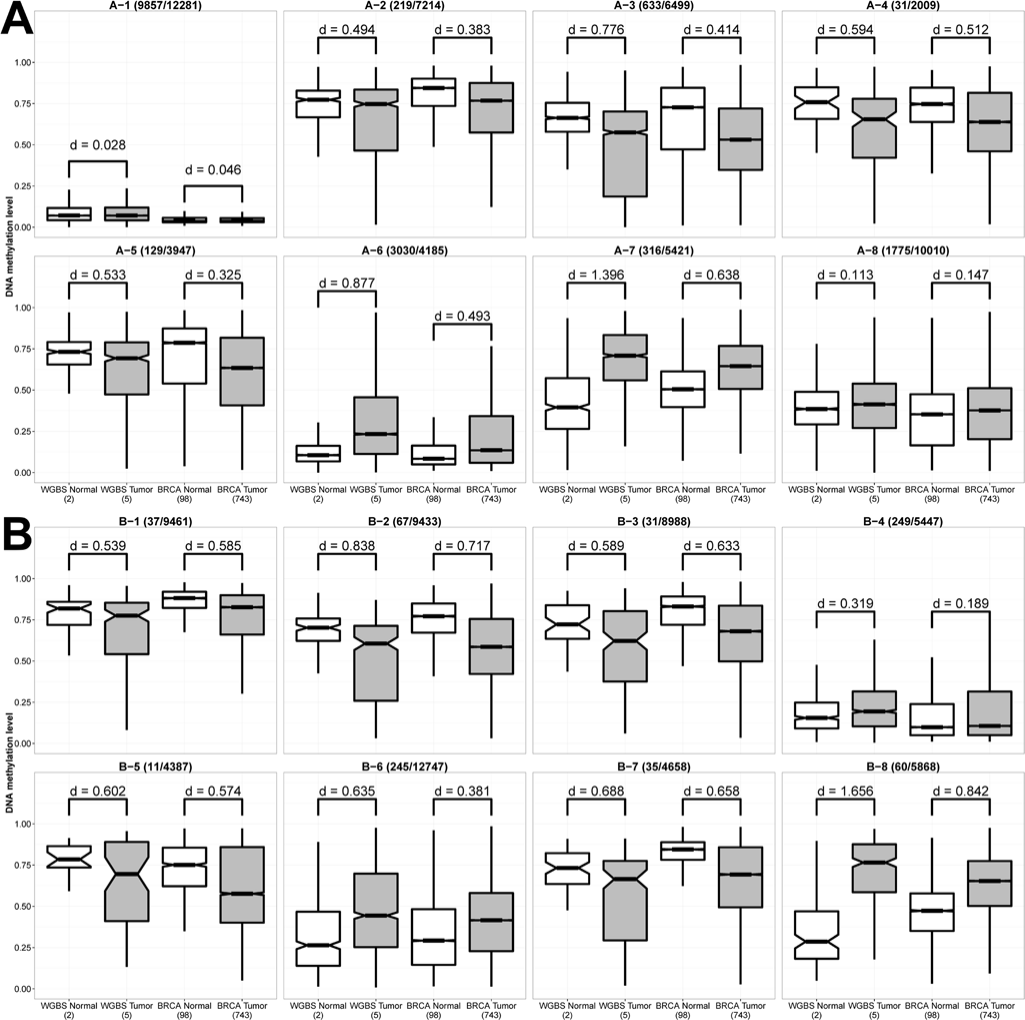
Difference in the distribution of DNA methylation levels of HMRs between normal breast and breast tumor samples in WGBS and TCGA BRCA datasets. The number of HMRs that had sufficient coverage of CpG sites on HumanMethylation450 BeadChip that were used in the plots and the total number of HMRs were provided beside the HMR cluster ID. The effect size for the difference between normal and tumor samples is provided as the d value. General consideration of small, medium and large effect has d values of 0.2, 0.5 and 0.8 respectively. (A) Promoter HMRs. (B) Intragenic HMRs.

We calculated the methylation levels at the selected HMRs for the 841 BRCA samples and the effect size for the difference between normal and tumor samples was assessed using the Cohen’s d method [43]. Similar to the WGBS samples, the A-1 HMRs were lowly methylated in the normal and tumor BRCA samples (d = 0.046) (Fig. 4A). We observed decreasing methylation levels at A-2, A-3, A-4 and A-5 promoter HMRs in tumor samples, but had small to median effect sizes (d = 0.3 to 0.5). Cancer-specific DNA hypomethylation is more significant in the intragenic regions, for example the B-1, B-2, B-3, B-5 and B-7 HMRs (d = 0.5 to 0.7), suggesting that DNA hypomethylation at the intragenic regions is a general phenomenon in primary breast cancer cells (Fig. 4B). The aberrant DNA hypermethylation of A-6, A-7 and B-8 HMRs seen in WGBS tumor datasets was also observed in the BRCA tumor samples (d = 0.5 to 0.9). IPA showed A-6 HMRs was enriched with genes associated with breast or ovarian cancer (p-value = 1.27E-10) (S8 Table).

### Discovery of over-expressed genes that exhibit promoter hypermethylation

**Fig 5:**
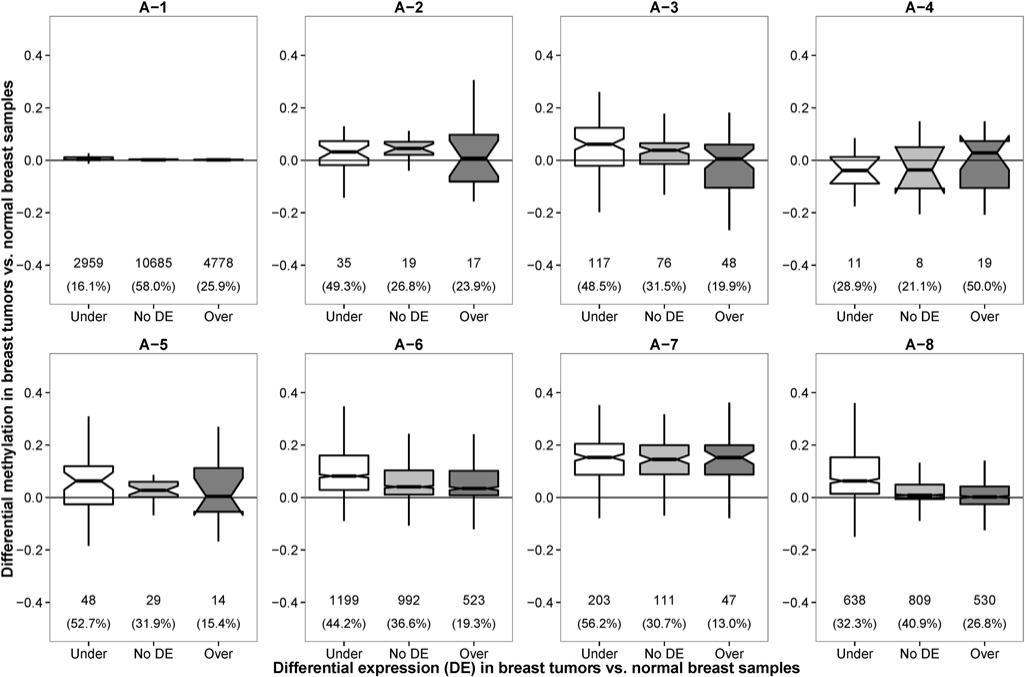
Distribution of differential methylation levels of gene promoters for under-expressed, over-expressed and not differentially expressed genes between breast cancer samples and normal breast samples of the TCGA BRCA datasets. The differential methylation and differential gene expression between breast cancer cell lines and normal samples were negatively-correlated in A-3, A-6 and A-8 HMRs as observed in the WGBS samples (Fig. 3).

We analyzed the correlation between promoter differential methylation and differential mRNA expression of the 113 BRCA paired tumor-normal RNA-seq data. Similar to that observed in WGBS datasets (Fig. 3), negative correlation between DNA methylation and gene expression were observed in A-3 and A-8 HMRs (Fig. 5). Also, majority of the promoters were associated with A-1 HMRs, and showed no differential methylation between normal and tumor cells. Despite the lack of differential methylation, many genes in A-1 were found to be significantly differentially expressed (such as *BAX*, *E2F1*, *FADD*, *GADD45A*, *PRKCA* and *TP53BP2* of the p53 signaling pathway) indicating that DNA methylation is probably not the key epigenetic regulator for these genes in breast tumors. Seven over-expressed breast cancer genes, namely *BCAR1*, *HSD17B1*, *MMP14*, *PLAU*, *SFRP2*, *SPP1* and *VCAN*, had increased promoter methylation and their promoters contained the tumor-specific hypermethylated A-6 or A-7 HMRs. Also, four over-expressed TSGs (*rap1GAP*, *THY1*, *GPR68* and *HOPX*) had differentially hypermethylated promoters in the tumors. These results implied that these genes may be negatively regulated by repressors in normal cells but were blocked by DNA methylation in tumor cells.

Analysis of the WBGS and RNA-seq also revealed five genes that were highly expressed and yet displayed complete DNA methylation near and within the genes (S3A to E Fig.). Four of them are non-coding RNAs that were highly expressed in both the HMEC and HCC1954 cells, and they are *U1* (snRNA), *SCARNA7* (scaRNA), *SCARNA9L* (scaRNA) and *SNORD71* (snoRNA). The fully methylated protein-coding *PPP2R2D* was moderately expressed in NB and MCF7 cells (S4 Fig.). In all five cases, the methylation of the genes and adjacent sequences did not appear to silence gene expression.

### Dysregulation of X-chromosome inactivation (XCI) in breast tumors

**Fig 6:**
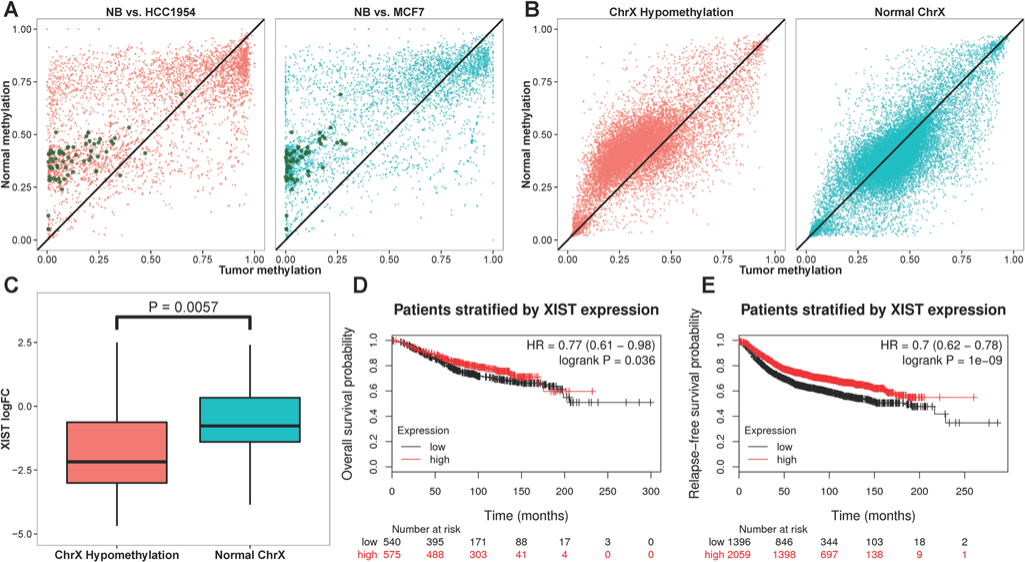
Aberrant promoter hypomethylation of genes located on the X chromosome in breast cancer cells. (A) Scatter plots of DNA methylation levels of NB (x-axis) and MCF7 and HCC1954 (y-axis; red and blue colors respectively) at promoter regions (TSS ± 1 Kb). The promoter regions that exhibited differential hypomethylation and associated with significant elevated downstream transcript expression (FDR ≤ 0.05) were colored in green. (B) Paired tumor-normal methylation assays of the TCGA BRCA dataset showed two populations of breast cancer patients where one group (colored in red) displayed promoter hypomethylation in the paired tumor samples as compared to the paired normal samples. (C) The group of BRCA patients that showed promoter hypomethylation had significantly reduced expression level of *XIST* in the corresponding paired tumor samples (t-test, p-value < 0.01). The (D) overall survival and (E) relapse-free survival analysis showed breast cancer patients with lower *XIST* expression had lower survival probabilities.

The A-8 HMRs were enriched with X-linked genes as described above. This group of HMRs exhibited varying degrees of abnormal DNA hypomethylation in breast tumors, and the change in DNA methylation and gene expression is negatively correlated (Fig. 2 and Fig. 3). We compared the DNA methylation of X-linked gene promoters in normal breast and breast cancer cell lines and found the differential hypomethylation pattern of MCF7 and HCC1954 is similar to that in several male methylomes (Fig. 6A and S5 Fig.). We suspected both breast cancer cell lines were lacking X-chromosome inactivation (XCI). Examination of the DNA methylation status of the *cis*-acting non-coding RNA *XIST*, that is both necessary and sufficient for initiating XCI [44, 45], showed that its promoter was methylated at 50% level in NB and HMEC, implying allelic methylation of *XIST*. In contrast, the *XIST* promoter was fully hypermethylated in MCF7 and HCC1954. RNA-seq data confirmed *XIST* was highly expressed in NB and HMEC but silenced in MCF7 and HCC1954. The aberrant silencing of *XIST* in MCF7 and HCC1954 was consistent with the observation of promoter hypomethylation and over-expression of 54 X-linked genes. Among these, 15 genes have been previously reported as XCI escapees (S9 Table) [46]. These results, together with the fact that *XIST* is only expressed from the inactive X chromosome, suggest that XCI is abolished in MCF7 and HCC1954.

Analysis of the BRCA dataset, containing 78 paired tumor-normal DNA methylation and RNA-seq data, showed 36 breast cancer patients exhibiting significant promoter hypomethylation of X chromosome gene in the tumor tissues as compared to the corresponding normal tissues (Fig. 6B). The expression of *XIST* RNA were also significantly lower in the paired tumor samples of this group of patients (t-test p-value < 0.01) and the overall and relapse-free survivals are lower for breast cancer patients with lower expression of *XIST* (Fig. 6C to Fig. 6E). From the 36 breast cancer patients, we identified 29 genes on the X chromosome to have significantly hypomethylated promoters and increased mRNA expression as compared with patients with relatively normal promoter methylation (t-test p-value < 0.01; S10 Table). Among these genes, *EBP*, *HAUS7*, *MED12*, *MORF4L2*, *MSL3*, *RPL10*, *SEPT6*, *TAZ* and *ZC4H2* have been shown to escape XCI [46]. Breast cancer survival analysis demonstrated that patients with high expression of *EBP*, *FAM127B*, *HPRT1*, *HTATSF1*, *MORF4L2*, *MOSPD1*, *PSMD10*, *SMS* or *TIMM8A* have lower overall and relapse-free survival, and high expression of *HAUS7*, *IDH3G*, *PL36A*, *SLC10A3*, *SLC9A6* or *UXT* have lower relapse-free survival.

### Identification of HMR clusters with high regulatory potentials in breast cancer methylomes

**Fig 7:**
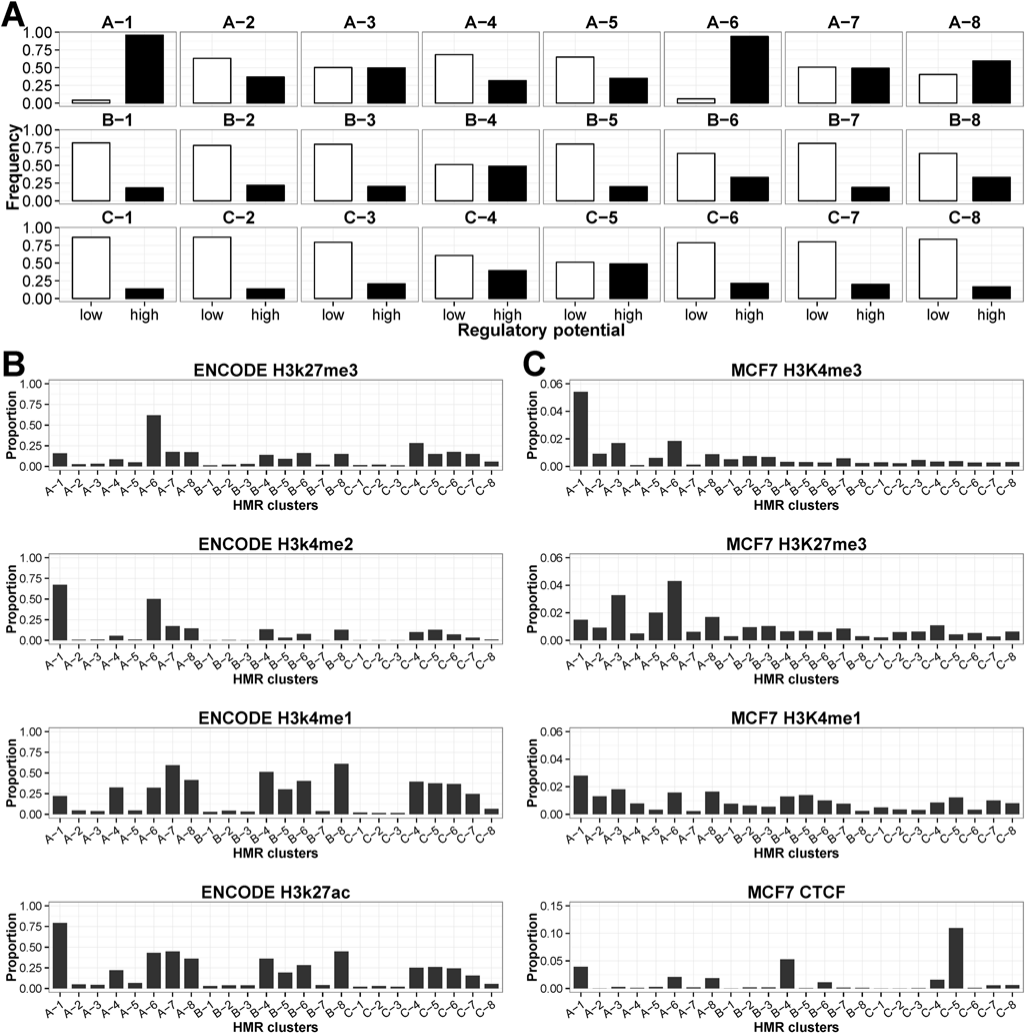
Association of HMRs with *cis*-elements and various histone modifications. (A) Proportion of different clusters of HMRs with “low” and “high” regulatory potentials. “Low” potential denotes HMRs that intersect less than four regulatory features, whereas “High” potential HMRs are those that intersect four or more features. A total of eight features were used in the analysis, they are: FANTOM5 TSS, FANTOM5 enhancers, CGI, ENCODE TFBS, ENCODE DNase I hypersensitive sites, MCF7 MNase hypersensitive sites. MCF7 HpaII hypersensitive sites, and RNA PolII binding sites from MCF7 and ENCODE. (B) Proportion of HMRs in each cluster that harbored high levels of ENCODE H3K27me3, H3K4me1, H3K4me2 and H3K27ac. (C) Proportion of HMRs in each cluster that harbored high levels of H3K27me3, H3K4me1, H3K4me3 and CTCF in MCF7. For each histone modification, the HMRs whose score is in the top 20% were considered as having high levels.

Change in promoter CGI methylation has been widely associated with mammalian transcriptional regulation. With the purpose of investigating the regulatory significance of HMRs, we compared the genomic locations of HMRs with eight public and in-house generated datasets to evaluate the regulatory potential of these regions. The features analyzed are TSS and enhancers from FANTOM5, CGI, ENCODE transcription factor binding sites (TFBS), ENCODE Deoxyribonuclease I (DNase I) hypersensitive sites, MCF7 micrococcal nuclease (MNase) and HpaII hypersensitive sites, and RNA polymerase II (PolII) binding sites from MCF7 and ENCODE (S6 Fig.). Fig. 7A showed ~90% of A-1 and A-6 HMRs overlaps four or more of regulatory features (denoted by “High” regulatory potential). The A-1 HMRs were associated with high level of H3K4me2, H3K4me3, H3K27ac and H3K9ac across eight ENCODE cell lines, and high level of H3K4me3 and H3K9ac in the ChIP-chip assay conducted in MCF7 (S7 Fig. and S8 Fig.). These showed genes associated with A-1 HMRs contain active epigenetic characteristics. In MCF7, the ChIP-seq experiments showed frequent co-occurrence of RNA PolII binding sites and A-6 HMRs. The A-6 HMRs were also strongly associated with the negative epigenetic mark, H3K27me3, as well as the positive histone marks such as H3K4me2 and H3K4me3, suggesting that they were associated with poised or bivalent promoter regions (Fig. 7B and Fig. 7C).

The remaining HMR clusters have moderate to low regulatory potential where they were CGI-poor and associated with fewer RNA PolII binding in MCF7. The A-7, B-4, B-8, C-4 and C-5 HMR clusters were enriched with FANTOM5 enhancers and ENCODE enhancer marks H3K4me1 and H3K27ac across eight ENCODE cell lines, indicating that they may represent active or inactive/poised enhancers in different cell types or developmental states (S6G Fig., S7A and E Fig.). Examples of known enhancers that displayed DNA methylation changes in these clusters include the tumor-specific hypomethylation of the distal enhancer of *MYC* (C-5 HMR) that is 67 Kb upstream of the TSS [47], and the hypermethylation of the enhancer in the second intron of *NOTCH1* (A-7 HMR) [48] (S9A and B Fig.). Differential hypomethylation of known regulatory elements such as the DNA replication initiation site located in the first intron of *DNMT1* gene (A-8 HMR) [49] and the estrogen receptor binding sites within intron 2 of *SLC22A5* (B-5 HMR) [50] were also observed in tumor cells (S9C and D Fig.). The intragenic B-4 and intergenic C-5 HMRs had low methylation levels across all seven samples as indicated in Fig. 2B and Fig. 2C respectively. They have the highest proportions of enriched CTCF signals in MCF7, and thus may contain regions involved in CTCF-dependent chromatin insulation (Fig. 7C).

### The switching between enhancer and heterochromatic state of the chromatin in normal and tumor cells

We made use of the published ChromHMM models for HMEC and MCF7 cells to determine the relationship between HMR clusters and the nine types of chromatin states [51] (S10 Fig.). In both cell types, around 60% and 20% of the active promoter states intersects the A-1 and A-6 promoter HMRs respectively, while B-4 and C-5 HMRs were enriched with CTCF sites. Both findings were consistent with the observations presented above. Approximately 17% and 6% of the poised promoter and repressed states in HMEC and MCF7 respectively were associated with A-6 HMRs.

The enhancer and heterochromatin were the two states that showed complementary association with the HMRs. The enhancer states were enriched with the HMR clusters: A-4, A-7 and A-8 promoter HMRs, B-4, B-5, B-6 and B-8 intragenic HMRs, and C-4, C-6 and C-7 intergenic HMRs identified by fisher’s exact test with multi-test adjustment. Comparing with MCF7, the HMEC cells have more enhancer states overlapping with A-7, B-8 and C-6 HMRs, and fewer heterochromatin states in these HMRs (S10 Fig.). Conversely, the A-4, B-5 and C-7 HMRs were enriched with MCF7 enhancers but few heterochromatin states. Hence, enhancers are important sites that exhibit methylation dysregulation in cancer cells, presumably through the dissociation and reassembly of heterochromatin structure.

**Fig 8:**
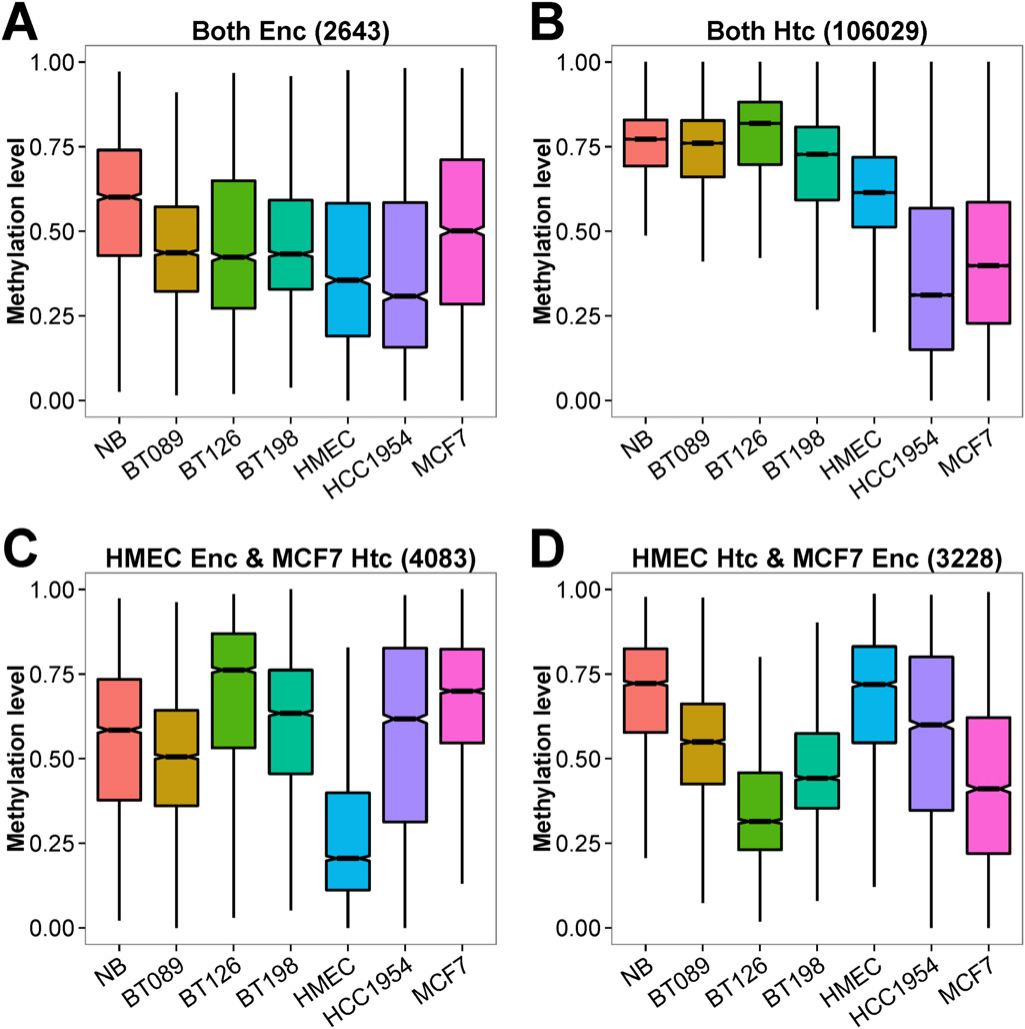
Distribution of DNA methylation levels of MCF7 and HMEC enhancers and/or heterochromatins in the seven breast methylomes. The genomic regions characterized as enhancer (Enc) or heterochromatin (Htc) in HMEC and MCF7 by ChromHMM were re-annotated as regions that were (A) enhancer in HMEC and MCF7 (B) heterochromatin in HMEC and MCF7, (C) enhancer in HMEC and heterochromatin in MCF7, and (D) enhancer in MCF7 and heterochromatin in HMEC.

We studied the DNA methylation of the HMRs associated with the enhancer and heterochromatin states in the HMEC and MCF7 cells (Fig. 8). The HMRs at regions that were classified as enhancers in both normal and tumor cells were moderately methylated in all seven WGBS datasets (median % methylation = 44%), although NB exhibiting higher DNA methylation level. In contrast, the HMRs marked as heterochromatic in both HMEC and MCF7 were highly methylated in normal cells and primary tumors (median % methylation = 77%). The cell lines (HMEC, MCF7 and HCC1954) showed unusual hypomethylation at these heterochromatic regions. As expected, the DNA methylations of HMRs at HMEC-specific enhancers were much lower in HMEC compared with other methylomes (median % methylation of 21% vs. 63%). At MCF7-specific enhancers, the HMRs were highly methylated in NB and HMEC, and lowly methylated in MCF7 (median % methylation of 72% vs. 39%). These MCF7-specific enhancers were also found to be moderately to lowly methylated in BT089, BT126 and BT198, suggesting that these regions may also be enhancers in primary breast tumors. In many aspects of our analysis, the benign fibroadenoma sample (BT089) and NB have highly similar DNA methylation profiles. Therefore, we believe the observation of DNA hypomethylation at MCF7-specific enhancers in the BT089 is a significant finding. The epigenetic abnormality at enhancer sites common in benign breast lesion and malignant breast tumors may possibly be disease-associated and potentially serve as markers for early diagnosis.

Large partially methylated domains (PMDs) are prominent hallmarks of epigenetic dysregulation of breast cell lines and tumors

**Fig 9:**
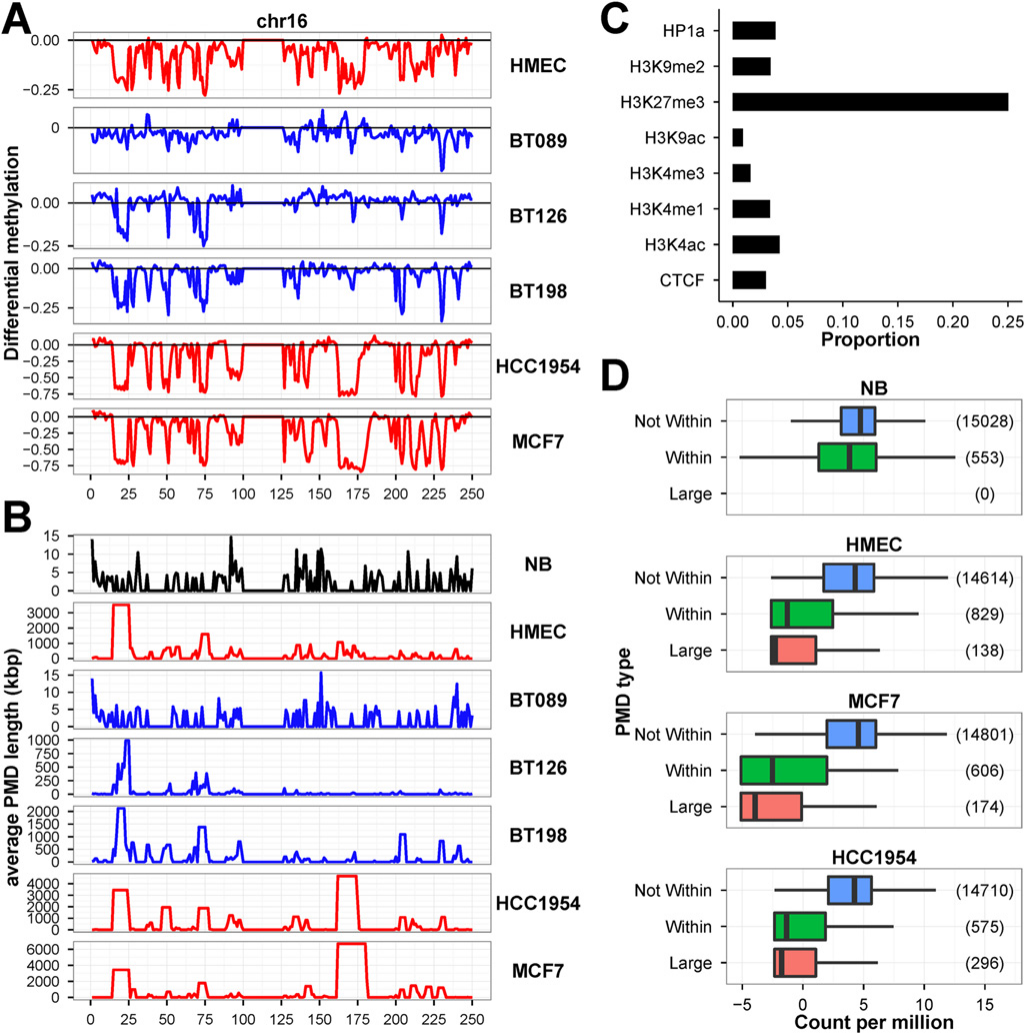
Hypomethylation of megabase-sized PMDs in breast tumors and cell lines. We used chromosome 16 as an examples to illustrate the (A) extent of differential methylation between NB and the other six WGBS datasets, and (B) average predicted PMD size (Kb) along the chromosome. (C) ChIP-chip assay in MCF7 showed PMDs were specifically enriched with H3K27me3 modification. (D) RNA-seq experiments showed genes located within PMDs and extremely large PMDs (> 1 Mb) have very low expression (represented as counts per million, CPM) than those outside PMDs.

In Fig. 1, we showed that invasive breast tumor cells and cancer cell lines exhibited extensive DNA methylation changes. Using the HMEC and MCF7 ChromHMM data, we estimated the heterochromatin constitutes approximately 80% of the genome and we showed, in Fig. 8, that cell lines displayed unusual DNA hypomethylation at heterochromatin. Such are the features of hypomethylated partially methylated domains (PMDs) in cancer cell lines [38, 40, 52, 53]. We identified between 2,600 to 4,200 PMDs in the seven methylomes (S11 Table). The PMDs cover less than 1% in NB and BT089, 7% in BT126 and 16% in BT198. In HMEC, MCF7 and HCC1954, the PMDs covered 25% to 35% of the genome. We used chromosome 16 as an example to demonstrate the varying degree but congruent DNA hypomethylation in immortalized cell lines and tumors along the chromosome (Fig. 9A). The broad valleys of large differential hypomethylated regions correspond to the PMDs in each methylome (Fig. 9B). The primary tumor samples (BT089, BT126 and BT198) had less hypomethylated PMDs than cell lines (HMEC, MCF7 and HCC1954). Nonetheless, the locations of PMDs are fairly consistent among the tumor cells. The cell lines harbored wider PMDs than primary tumors, probably as a consequence of lagging DNA methylation due to accelerated cell growth [38]. Although the immortalized breast cell lines and primary breast tumors had varying degrees of hypomethylation and PMD sizes, they appeared to have shared properties at many PMDs. Overall, the PMDs are associated with regions of lower CpG density, gene deserts or large tissue-specific genes, and with the lamin B1 which is an indication of the close proximity of PMDs with nuclear envelop (S11 Fig.). In MCF7 cells, the fluorescent *in situ* hybridization assay using the McrBC-resistant fragments confirmed that the large hypomethylated DNA was indeed located at the nuclear periphery (S12 Fig.). S13 Fig. showed that the known fragile site loci were strongly associated with PMDs and PMD-containing fragile sites were significantly hypomethylated in the advanced breast tumors and breast cell lines. Therefore, the hypomethylation of the PMDs in breast tumor also have implications in genomic instability and tumorigenesis.

With the ChIP-chip assays performed on MCF7, we found PMDs are most associated with the repressive polycomb-associated H3K27me3 mark (Fig. 9C). By analyzing the RNA-seq expression data, we showed that genes located outside PMDs had higher expression than those within PMDs in the cell line samples (Fig. 9D). Genes that are located in extremely large PMDs (> 1 Mb) had the lowest expression values.

## Discussion

In this study, we performed WGBS to uncover the DNA methylation landscapes of normal breast, primary breast tumor cells and MCF7 breast cancer cell line. Unlike the widely used microarray platform that has limited CpG coverage, WGBS allowed us to detect DNA methylation at single-base-resolution, and identify differential methylation at focal regions (i.e. HMRs) and large zones (i.e. PMDs).

The HMRs are hypomethylated regions that usually co-locate with CGI and span both the CGI and the surrounding CGI shores. In HMRs that were conserved between NB and other breast methylomes, we repeatedly observed the expansion at CpG-poor regions and contraction at CpG rich regions in invasive tumor cells and cancer cell lines. The increasing hypomethylation of the intragenic regions and hypermethylation of the CGI-associated promoter in breast tumor cells are consistent with previous cancer methylomic studies [18, 20].

An interesting observation in this study is the subtle expansion/contraction of HMRs in the benign tumor BT089. There are 27 tumor suppressors and 304 transcription factors associated with the altered promoter HMRs. The two significantly up-regulated TSGs, *ST14* and *SFRP4*, are genes known to be involved in mesenchymal to epithelial transition (MET). Indeed, the MET regulators (*OVOL1*, *OVOL2*, *IRF6*, *ESRP1* and *ESRP2*) and epithelial markers (*CDH1*, *KRT8*, *KRT18*, *ST14*, *PRSS8*, *DSP*, *OCLN*, *SCNN1A*, *SPINT1*, *SPINT2* and *TJP3*) were over-expressed in BRCA tumors as compared with normal tissues. On the other hand, the epithelial to mesenchymal transition (EMT) regulators and mesenchymal markers (*ZEB1*, *ZEB2*, *TWIST1*, *NOTCH1*, *DCLK1*, *DCN*, *LIX1L*, *PMP22*, *SNAI2*, *SOX10*, *TCF4*, *TSHZ1* and *VIM*) are under-expressed in BRCA tumors. The over-expression of key epithelial markers correlated with increased HMR widths observed in BT089, suggesting that epigenetic regulation through change in DNA methylation also played a part in the expression of epithelial phenotypes. Our finding that early stage tumors expressed MET markers is consistent with the role of MET in tumor expansion, where EMT can promote cancer stem cell properties, tumor invasion, and resistance to chemotherapy, and MET results in increased cell proliferation and promote metastases [54, 55]. Nonetheless, the EMT-associated *HDAC2* and *EPN3* were differentially hypomethylated in BT089 and up-regulated in “Stage 1” BRCA tumors. Moreover, the EMT-inhibiting *EHF* and *TP63* were down-regulated and the cancer invasion-associated *EPSTI1* was up-regulated implying that proliferation of mesenchymal elements and may be important in the epithelial plasticity and cancer progression.

We have identified differentially methylated HMRs clusters that may be enhancers in breast tumors, as well as breast cancer genes with differential gene expression. Our analysis also revealed the dysregulation of XCI in breast cancer cell lines as well as a subset of primary tumor tissues in the BRCA dataset. In MCF7 and HCC1954, the unusually hypomethylation of a large numbers of promoters on the X chromosome have transformed their X chromosome DNA methylation patterns to that of male patterns (such as H1 and HUES64). Coupled with the fact that *XIST* is not expressed in both MCF7 and HCC1954, the data suggest loss of XCI, and may result in up-regulation of oncogenes on the X chromosomes, down-regulation of TSGs and increased cell proliferation [56, 57]. Indeed, the promoters of several breast cancer genes on the X chromosome, such as *AR*, *HMGB3* and *LAGE3*, were hypomethylated and the mRNAs were over-expressed in MCF7 and HCC1954. Analyses of the BRCA dataset showed that aberrant hypomethylation of X-linked genes occurred in nearly 50% of breast cancer samples with correspondingly reduced *XIST* expression. The over-expression of several promoter hypomethylated genes is associated with lower survival of patients with breast cancer. Hence, the defects in maintaining proper DNA methylation of the X chromosomes could play a role in the development and progression of breast cancer. Given that patients with dysregulation of X-linked genes showed poor clinical outcome, these genes could serve as useful markers for breast cancer. We believe the methylome clustering analysis may provide a useful tool for uncovering novel genes and regulatory elements involved in breast tumorigenesis.

Hierarchical clustering also identified the universally lowly methylated A-1 HMRs that are associated with CGI-containing housekeeping genes and active promoter marks. These genes are in an active state and showed no differential expression between normal and tumor samples irrespective of their expression levels. The results indicate that there are functional, physical and possibly selective constraints that prevent these regions from epigenetic changes during tumorigenesis. Furthermore, the absence of substantial differential methylation in the CGI-rich promoters of housekeeping genes opens up the possibility of revamping the DNA methylation microarray probe design algorithms.

Unlike previous belief [58], recent genome-scale sequencing data has unveiled the fact that change in promoter DNA methylation is not always a reliable predictor of differential gene expression [52]. Our findings showed many genes that displayed significant differential expressions had very few DNA methylation changes at promoter regions. The over-expressed *CCNE1*, *CLGN*, *NEK2*, *PTTG1* and *RAD51*, and the under-expressed *ADAMTS1*, *FOS*, *FOSB*, *IL6* and *ZFP36* in tumor cells are examples of breast cancer genes without differential promoter methylation between normal and tumor samples. Similarly, we also observed genes that were hypermethylated at promoter regions but expressed in breast tumors (such as *HOPX* and *THY1*). The *HOPX* and *THY1* are well-studied TSGs in colorectal, ovarian and nasopharyngeal cancers [59, 60]. Similar to the findings from those studies, both genes were completely silenced in the MCF7 and HCC1954 attributed to complete promoter DNA hypermethylation. Therefore it is surprising to observe increased expression of *HOPX* and *THY1* in the BRCA datasets that exhibited increased promoter DNA methylation (of the promoter CGI or CGI shores). We postulate that the hypermethylation observed in breast tumors only affects the repressor binding site and block a repressor from binding, hence the usual dampening of transcriptional activity were lifted. Unlike the complete methylation of the promoters in MCF7 and HCC1954, the remaining of the promoter region stays hypomethylated to enable transcription in breast tumors. Other studies have hypothesized DNA methylation being a secondary event whereby other mechanisms of transcriptional regulation have already taken place to silent or activate genes [42, 61]. In the cases of p53 and AP1, both transcription factors were not sensitive to methylation, DNA methylation may participate in the chromatin remodeling to indirectly block access of these proteins to their cognate binding sites [14].

Another genome-wide irregularity in the epigenetic control is the formation of large hypomethylation zones in tumor cells. We previously showed that the PMDs are unique features in cancer cell lines from various tissue origins using biochemical methods [62, 63]. The PMDs that span over 5 Mb in length are cell line-specific and were found in both the normal and cancer cell lines. Hon *et al* had suggested the formation of PMDs was the result of gradual loss of DNA methylation through successive cell divisions in actively replicating cells [38]. PMDs are associated with heterochromatin that is usually replicated late in cell cycle; hence aberration of DNA methylation sets in near the end of cell cycle in actively replicating cells. The PMDs identified in the invasive breast primary tumors were shorter but their locations usually coincide with the wider PMDs identified in breast cell lines. This observation suggests that the size and number of PMDs in primary tumors could serve as an indication of tumor proliferation activity. The emergence of PMDs at CpG-poor gene deserts, large tissue-specific genes and the association with repressive mark H3K27me3 and nuclear periphery are common properties shared by immortalized cell lines and invasive primary tumors [38, 40]. The megabase-sized demethylation over the entire lengths of genes has repressive effect on gene expression in cancer cell lines [36]. Besides PMDs, we also found systematic intragenic DNA hypomethylation (in the form of intragenic HMRs) in breast tumors. Hence, the global reduction of DNA methylation in cancer cells is the result of DNA hypomethylation at both HMRs and PMDs. Given that PMDs in breast tumors are found in chromosome fragile sites, the aberrant methylation of these sites could cause genomic instability which is a hallmark of cancer [64].

## Conclusion

Our study explores the DNA methylation landscapes of normal and breast cancer cells by using WGBS. Our results revealed the extent of methylation changes in cancer cells and confirmed the significance of differentially methylated HMRs in breast cancer. We showed the widening of HMRs in fibroadenoma corresponds to the over-expression of MET transcription factors and epithelial markers in tumor cells of early-stage breast cancer patients. By performing hierarchical clustering of reference HMRs using the methylation levels of each methylome as their distance, we characterized the aberrantly hypermethylated regions that are highly associated with breast cancer. The results are consistent with our analyses of the TCGA BRCA methylation dataset that has a larger pool of breast cancer patients. Our study provides further evidence that DNA hypomethylation of intragenic and intergenic regions and the occurrence and widening of PMDs are common features of breast tumor cells. Furthermore, the impairment in the maintenance of XCI may have the capacity to influence breast cancer epigenome.

## Materials and Methods

### Cell culture

MCF7 cells, originally obtained from ATCC (Manassas, VA), were cultured in RPMI1640 medium (GIBCO/BRL) supplemented with 10% (v/v) fetal bovine serum (GIBCO/BRL), 2.0 g/L sodium bicarbonate, and were incubated in a humidified 37°C incubator with 5% CO_2_.

### Genomic DNA extraction

MCF7 cells were washed with 1X PBS and resuspended with cell lysis buffer. Cells were treated with 0.1 mg/mL of RNaseA for an hour at 37°C and 0.3 mg/mL proteinase K for 12–16 hours at 55°C. DNA was extracted with an equal volume of phenol/chloroform/isoamyl alcohol mixture (24:25:1). The extraction procedure was repeated until the interface is clean. An equal volume of chloroform was then added and the mixture centrifuged for 10 minutes at 13000x g. Finally, the aqueous phase was removed and precipitated with ethanol. After removal of the supernatant, the DNA pellet was washed with 70% ethanol, air-dried, and dissolved in triple distilled H_2_O. The integrity of the DNA extracted was checked by 1.2% (w/v) agarose gel electrophoresis. The concentration of DNA was estimated by ultraviolet spectrophotometry.

### Preparation of RNA

MCF7 cells were grown to 85% confluence in 6 cm tissue culture dish. Each 6 cm dish was rinsed twice with 1X PBS. Total RNA was extracted using TRIreagent (Invitrogen) protocol. The integrity of the RNA extract was checked by 1.2% (w/v) agarose gel electrophoresis and the concentration of RNA was estimated by ultraviolet spectrophotometry.

### Human tissue genomic DNA and RNA

The following genomic DNA of human adult normal breast and tumor tissues were purchased from BioChain (Hayward, CA): normal breast (Catalog No.: D1234086, Lot No.: B503025). BT089 (Catalog No.: D1235086-DC, Lot No.: A805089), BT126 (Catalog No.: D1235086, Lot No.: A808126) and BT198 (Catalog No.: D1235086, Lot No.: B410198). The normal breast tissue mRNA were purchased from Origene (Catalog No.: CR559104 and CR561898)

### Chromatin immunoprecipitation (ChIP)

Immunoprecipitation was performed according to the manufacturer’s protocol (Upstate Biotechnology, Inc., Lake Placid, NY) with slight modifications. MCF7 cells were fixed for 10 min with 1% of formaldehyde at room temperature, and then quenched with a final of 1M of glycine. The cells were lysed and sonicated to shear DNA to a length of 200–500 bp with a Bioruptor (Diagenode, Sparta, NJ). Lysates were pre-cleared with protein A-agarose beads and targeted chromatin was immunoprecipitated with antibodies against H3K4me1 (ab8895; Abcam), H3K4me3 (04–745; Millipore), H3K4ac (07–539; Millipore), H3K9ac (06–942; Millipore), CTCF (sc-15914X; Santa Cruz Biotechnology), H3K27me3 (ABE44; Millipore), H3K9me2 (07–212; Millipore), HP1a (05–689; Millipore) and RNA polymerase II (ab5408–100; Abcam). The beads were washed once with each washing buffer, including low salt immune complex wash buffer, high salt immune complex wash buffer, and LiCl immune complex wash buffer, and twice with 1X TE buffer. Precipitates were eluted with 1% of SDS and 100 mM of NaHCO_3_. The samples were heated at 65°C for 6 hours in order to reverse cross-link, extracted with phenol/chloroform, ethanol-precipitated.

### Array-CGH protocols

The ChIP samples were amplified according to the manufacturer’s protocol (GenomePlex Complete Whole Genome Amplification Kit, Sigma). The DNA samples were analyzed using Agilent Human CGH Microarray 1M (Agilent Technologies). DNA quality, sample labeling, hybridization and washing were performed according to the protocol provided by Agilent. Slides were scanned with an Agilent Scanner. The captured images were transformed to data with Agilent Feature Extraction software and the results were presented using Agilent CGH Analytics software. The Cy3 hybridization intensity was normalized to Cy5 for comparison among the samples. The log2 ratios (log2 Cy5/Cy3) were calculated and compared.

### Library preparation and sequence data generation

**Whole-genome bisulfite sequencing**. Bisulfite-seq library was prepared using a method based on Lister *et al* [32]. Briefly, 3–5 ug of DNA was fragmented by sonication with a Bioruptor (Diagenode, Sparta, NJ) following by adapter ligation using the Pair End DNA Sample Prep kit (Illumina Inc., USA) with the use of methylated adapter (Illumina Inc., USA) according to manufacturer’s instruction. For each sample, four adapter-ligated DNA fragments of 200–250 bp, 250–300 bp, 300–350 bp and 350–400 bp were isolated by gel electrophoresis and subjected to bisulfite conversion and PCR enrichment independently to generate four separate libraries for each sample. Bisulfite treatment was performed using an EZ DNA Methylation-Gold Kit (Zymo Research) that converts unmethylated cytosines to uracils and leaves methylated cytosines unchanged. Four separate PCR were performed for each library using PfuTurbo Cx Hotstart DNA polymerase (Stratagene) and then pooling the enrichment products following by gel purification. PCR-amplified library was quantified by quantitative PCR and the library size was determined on an Agilent 2100 Bioanalyzer with High Sensitivity DNA chip. Bisulfite-seq library was sequenced on a Genome Analyzer IIx or HiSeq2000 (Illumina Inc., USA) by paired-end sequencing with 100 or 150 nucleotide read length.

**mRNA sequencing**. The sequencing library for mRNA-seq was prepared using TruSeq RNA Sample Preparation Kit (Illumina Inc., USA) as per manufacturer’s instruction. Briefly, total RNA with RNA integrity number (RIN) greater than 7.5 was subjected for poly-A mRNA isolation using poly-T oligo-attached magnetic beads. The poly-A mRNA was fragmented and first-strand cDNA was synthesized using random hexamers following by second-strand cDNA synthesis, end repair, addition of a single A base and adapter ligation. The adapter-ligated cDNA library was sized-selected by agarose gel and amplified by PCR. The enriched RNA-seq library was sequenced on HiSeq2000 (Illumina Inc., USA) by paired end sequencing with 100 nucleotide read length.

**ChIP sequencing**. The sequencing libraries were constructed from immunoprecipitated and input DNA using TruSeq ChIP Sample Preparation Kit (Illumina Inc., USA) according to the manufacturer’s instruction. The fragmented DNA was end repaired following by addition 3’-A to the ends and ligation of adapters. The adapter-ligated DNA library was size-selected (300–500 bp) on a 2% agarose gel and amplified by PCR for 16 cycles with the use of KAPA HiFi DNA Polymerase (Kapa Biosystems). The enriched library was sequenced on a HiSeq2000 (Illumina Inc., USA) by single end sequencing with 100 nucleotide read length.

**MNase hypersensitive assay**. MCF7 cells were washed with ice-cold 1X PBS, and lysed with 700 μl of MNase lysis buffer for 15 min on ice. Cell nuclei were gently rinsed and suspended in 650 μl of MNase digestion buffer (with CaCl_2_). The reaction was performed by adding 5U of MNase and incubating at 25°C for 5 min. Reaction was terminated by adding 40 μl of MNase stop buffer and 20 μl of 20% SDS. Suspensions were collected and treated with 0.1 mg/ml of RNase A for an hour at 37°C, and then with 0.3 mg/ml of proteinase K for 12–16 hours at 55°C. DNA was phenol/chloroform extracted and ethanol-precipitated. The integrity of the DNA extracted was checked by 1.2% (w/v) agarose gel electrophoresis. The concentration of DNA was estimated by ultraviolet spectrophotometry.

The 454 sequencing libraries of MNase treated DNA were constructed using GS 20 DNA Library Preparation Kit (Roche Diagnostics) with the omission of any DNA shearing step and followed the recommended modification for low molecular weight DNA samples. Briefly, DNA fragments ends were polished and ligated with 454 adapters. The adapter-ligated DNA fragments were then immobilized onto streptavidin beads, repaired by a fill-in polymerase followed by alkaline denaturation to isolate single-stranded DNA (ssDNA) library. The quality and quantity of the ssDNA library was assessed using the Agilent 2100 Bioanalyzer. The ssDNA library was clonally amplified by emulsion PCR to enrich the fragments and following by pyrosequencing reaction run on a GS 20 (Roche diagnostics) with 100 nucleotide read length.

***In situ* HpaII digestion-based sequencing assay**. MCF7 cells were washed three times with ice-cold 1X PBS on culture dish. The cells were lysed with 700 μl of ice-cold TZN buffer (10 mM Tris-HCl pH 7.6, 0.2 mM ZnCl2, 0.2% NP-40) on ice for 15 min. The lysate was removed by suction, and then gently rinsed the nuclei with 700 μl of 1X NEB buffer 1. The reaction was performed by incubating cell nuclei in 650 μl of 1X NEB buffer 1 and 50U of HpaII at 37°C for 6 hours. To fill in and mark the DNA ends, 30 μM dATP, dGTP, dTTP, biotin-14-dCTP (Invitrogen), and 10 ul 5U/μl Klenow (NEB) were added to cell nuclei. The mixtures were incubated at 37°C for 1 hour and subsequently placed on ice. The reaction was terminated by added 40 μl stop buffer (100 mM EDTA and 10 mM EGTA) and 20 μl of 20% SDS. Suspensions were collected in a 1.5 ml Eppendorf tube and treated with 0.3 mg/ml of proteinase K for 12–16 hours at 65°C. Samples were extracted with equal volumes of phenol/chloroform/isoamyl alcohol mixture (24:25:1), the extraction procedure was repeated until the interface was clean. An equal volume of chloroform was then added, and the solution was centrifuged for 10 min at 13,000g. The aqueous phase was ethanol-precipitated, and the DNA pellet was washed with 70% ethanol, air-dried, and dissolved in d3H_2_O.

2–3 ug of HpaII treated and biotin labeled DNA was fragmented by sonication with a Bioruptor (Diagenode, Sparta, NJ) and size-fractionated by 2% agarose gel. Two biotinylated DNA fragments of 300–500 bp and 500–800 bp were purified by Dynal magnetic M-280 streptavidin beads and subjected to library construction independently by performing end-repair reaction, addition 3’-A to the ends and adapter ligation on the biotinylated DNA immobilized to the streptavidin beads with the use of TruSeq DNA Sample Preparation Kit (Illumina Inc., USA). Two separate PCR of 18 cycles were performed for each fragment libraries using KAPA HiFi DNA Polymerase (Kapa Biosystems) and then pooling the enrichment products followed by purifying with AMPure XP Beads. PCR-amplified libraries were quantified by quantitative PCR and the library size was determined on an Agilent 2100 Bioanalyzer. The libraries were sequenced on a HiSeq2000 (Illumina Inc., USA) by paired-end sequencing with 100 nucleotide read length.

### Next-generation-sequencing data analysis

**Whole-genome bisulphite sequencing**. The *fastq_masker* of the FASTX-Toolkit suite (v0.0.13) was used to convert nucleotides with quality score less than 30 to “N” before read mapping. The bisulfite sequencing reads were processed and analyzed using the MethPipe suite (v3.0.11) according to the protocols suggested in the MethPipe manual [65]. The hg18 human genome was used as the reference genome for mapping, allowed at most three mismatches, and estimated the length of the paired-end insert to about 400 bp. The *rmapbs-pe* was used for mapping and the *duplicate-remover* was used to remove duplicate reads due to PCR amplification and to extract unique mapped reads. The bisulfite conversion rates were calculated using *bsrate*, and *methcount* was used to call the methylation level of each CpG site. We used *hmr* to call for hypomethylated regions with 1 Kb maximum CpG distance, and *pmd* to identify partially methylated domains with 20 Kb maximum CpG distance. For visualization, the DNA methylation level is represented as a continuous value that ranges from 0 and 1 at each CpG site, denoting fully unmethylated and methylated respectively. To compare the methylation value between samples, we subtract these values at each common CpG site to produce a differential methylation value between-1 and 1, denoting differential hypomethylation and hypermethylation respectively.

**mRNA sequencing**. Sequencing reads were mapped to the human reference genome (hg18) using STAR (v2.3.0e)[66]. The R Bioconductor package edgeR [67] was used to identify differentially expressed genes between normal samples (NB and HMEC) and tumor samples (MCF7 and HCC1954) (FDR ≤ 0.05) using count data generated from *featureCounts* [68] of the Subread package based on the human coding sequence annotation (GENCODE v3c) GTF file downloaded from the GENCODE project website (http://www.gencodegenes.org).

**ChIP sequencing**. The Illumina 100 bp single end raw reads were aligned to the human reference genome (hg18) with bowtie2 (v2.0.6) [69]. Only uniquely mapping reads were used. Duplicated reads were removed with Picard tools (v1.92). The predominant insert-size (fragment length) was estimated using the phantompeakqualtools package [70]. The MACS (v2.0.10) [71] and Irreproducible Discovery Rate (IDR) framework [72] were used for peak calling from replicate experiments.

**MNase sequencing**. Roche 454 raw reads were mapped to the human reference genome (hg18) with BWA-MEM (v0.7.5a) [73]. Uniquely mapping reads and properly paired reads were selected for further analysis using the SAMtools utilities (v 0.1.19, http://samtools.sourceforge.net/). Duplicated reads were removed with Picard tools (v1.92, http://picard.sourceforge.net/). The processed reads from MNase-seq will map the sensitive regions in the incompletely digested chromatin samples.

**HpaII digestion-based sequencing**. Illumina 100 bp paired end raw reads were processed using the same protocol as MNase sequencing. After removing duplicated reads, the sequence reads was processed further as follow: (a) We identified all the HpaII cleavage sites in the human genome (~2.3 million sites in hg18). (b) We assigned the processed reads to the nearest HpaII cleavage site with *closestBed* in the BEDtools suite (https://github.com/arq5x/bedtools2). (c) The reads that were more than 10 bp away from the nearest HpaII cleavage sites were discarded. The processed read from HpaII-seq will map the flanking sequences of cleaved unmethylated CCGG sites in the incompletely digested chromatin samples.

### Data visualization

**Whole-genome bisulphite sequencing data**. To facilitate examination of the normal breast and tumor DNA methylomes, the CpG methylation profiles were visualized on UCSC Genome Browser. A score (range: -0.5 to 0.5) is given to each CpG site to represent the fraction of methylation with 0 being 50% methylation. Hypermethylated CpG (methylation > 50%) were represented with upward red bars, whereas hypomethylated CpGs (methylation < 50%) were represented with downward green bars.

**Other sequencing data**. For peak data, the BED files were converted to BigBED format with the *bedToBigBed* UCSC utility (http://hgdownload.cse.ucsc.edu/admin/exe/). For continuous-valued data, the BAM files were converted to BigWiggle files using *genomeCoverageBed* in the BEDtools suite and *bedGraphToBigWig* UCSC utility. These custom tracks were then visualized online using the UCSC Genome Browser Track Data Hubs.

#### Additional statistical and bioinformatics analyses

The HMRs from all samples were merged using *mergeBed* in the BEDtools suite to generate a reference set containing 174,439 regions. For each WGBS sample, the DNA methylation level of individual reference HMR was re-calculated. The *daisy* function (R package cluster) was used to compute all Gower pairwise dissimilarities of the HMR methylation values among WBGS samples. Then the *hclust* function (R package stats) was used to perform hierarchical clustering (Ward’s method). Finally, heatmaps were produced using the *heatmap*.*2* function (R package gplots).

The intersection (co-occurrence) and data summarization of the genomic features were calculated using *intersectBed* and *groupBy* of the BEDTools suite.

Breast cancer overall survival and release-free survival analysis was performed using Kaplan Meier-plotter [74]. Samples were stratified into high or low expression of genes selected for survival analysis using the “auto select best cutoff” option. The hazard ratio with 95% confidence intervals and log-rank P-value were estimated using the Cox proportional hazards (CoxPH) model (R package survival). Survival curves were produced using the survplot package of R environment.

Cohen’s d effect size analysis was used to determine the size of methylation difference between normal and tumor samples in units of standard deviations [43]. In general, the effect sizes are categorized as small (0.2), medium (0.5) and large (0.8) according to Cohen. The unpaired Student’s t-test was used to test for differences between two groups of continuous variables.

### mRNA expression levels of DNMTs by quantitative real-time PCR (qRT-PCR)

Reverse transcription was performed by using SuperScript III RNase H- Reverse Transcriptase (Invitrogen) with random hexamer according to the manufacturer’s protocol. Quantitative RT-PCR was performed using KAPA SYBR FAST (KAPA Biosystems, KK4603) on ABI StepOnePlus Real-Time PCR System. All reactions were performed in triplicate with KAPA SYBR FAST plus 10 μM of both the forward and reverse primers according to the manufacturer-recommended thermal cycling conditions, and then subjected to melting curve analysis. *PPP2R2D*, *SPDEF*, *PDGFRB*, *VCAN* and *ACTB* Ct values were normalized to 18S Ct values. Gene expression was determined using the delta-delta Ct method.

### Bisulfite conversion and bisulfite DNA sequencing

Bisulfite conversion was performed by using 1500 ng of purified genomic DNA with EZ DNA Methylation-Lightning Kit (Zymo Research) according to the manufacturer’s protocol. PCR primers were designed to amplify the designated promoter regions. The bisulfite primer sequences were listed in S12 Table. Following PCR amplification, gel-purified bands were cloned into the yT&A vector (Sigma). Approximately 10 individual clones from each PCR product were submitted for DNA sequencing. The sequences were trimmed to remove vector sequence and low quality sequences, and subsequently analyzed to evaluated the methylation status of the target CpG sites.

### Fluorescence *in situ* hybridization

MCF7 cells were fixed on the coverslips with 3.7% formaldehyde in PBS, permeabilized with 0.5% Triton X-100. The McrBC-resistant DNA probes were labeled with Texas Red by random prime (Invitrogen). Hybridization mixture in all samples consisted of 50% formamide, 10% dextran sulfate, 1X SSC (MM 1.0). For interphase FISH, a sufficient volume of probe was loaded onto coverslips with fixed and pretreated cells. A slide was used to cover an area with cells and sealed with rubber cement. Cells and probe DNA were denatured simultaneously on a hot-block at 76°C for 10 min. Hybridization was performed for 2 days at 37°C in the humid boxes. Post-hybridization washed were performed with wash solution (three times), 2X SSC (two times), and 1X SSC (one time) at 45°C, respectively. Nuclear DNA was counterstained with 0.5ug/ml DAPI, and cells were mounted in antifade medium. Slides were examined on a Leica TCS SP5 confocal microscope with 63X oil objective and the appropriate filters.

### External datasets

The UCSC Genes (knownGene), Ensembl Genes (ensGene), Gencode Genes (wgEncodeGencode), CpG Islands Tracks (cpgIslandExt), ENCODE Integrated Regulation Tracks (wgEncodeReg), ENCODE/Broad Institute Histone Modifications (wgEncodeBroadChipSeq), and NKI LaminB1 track (laminB1) were obtained from the UCSC genome browser (http://hgdownload.soe.ucsc.edu/). The bisulfite sequencing data for HMEC and HCC1954 were obtained from NCBI GEO (GSE29127) [38]. The processed bisulfite sequencing Wig files for breast luminal epithelial cells (UCSF-UBC), breast myoepithelial cells (UCSF-UBC), esophagus (UCSD), gastric (UCSD), H1 (UCSD), H9 (UCSD), HUES64 (BI), lung (UCSD), penis foreskin fibroblast primary cells (UCSF-UBC), penis foreskin keratinocyte primary cells (UCSF-UBC) were obtained from the NIH Roadmap Epigenomics data listings at NCBI GEO (http://www.ncbi.nlm.nih.gov/geo/roadmap/epigenomics/). The ChIP-seq data for MCF7 RNA PolII binding profile was obtained from GEO (GSE32692). The ChromHMM classification in HMEC and MCF7 cells were obtained from GEO (GSE57498) [51]. The processed “Level 3” DNA methylation and RNA-seq data of TCGA breast invasive carcinoma (BRCA) dataset were obtained from the TCGA Data Portal (https://tcga-data.nci.nih.gov/tcga/) (S13 Table). The fragile sites data were obtained from the GENATLAS database (http://www.dsi.univ-paris5.fr/genatlas/). The CrossMap (v0.1.4) tool was used to convert hg19 coordinates in the BED and Wig files to hg18 coordinates and to perform format conversion when necessary [75].

## Supporting Information

S1 Fig HMR properties. (A) We compare the size of normal breast (NB) HMRs and the intersected CGI and showed HMR are generally wider than CGI. This information implied CGI shores, regions directly flanking CGI, are also hypomethylated. (B) The HMR sequences that directly overlapped CGIs and CGI shores were both associated with high number of TFBS compared with randomly selected regions from human genome. (C) The HMRs identified in NB were compared with those in primary breast tumors (BT089, BT126 and BT198) and breast cell lines (HMEC, MCF7 and HCC1954). In the x-axis, the plus (+) symbol denotes expansion and minus (–) symbol denotes contraction of the NB HMRs. HMRs that had log2 fold change between 0.19 ~ 1 have small changes in widths (+ or –); log2 fold change between 1 ~ 3 have large changes (+ + or – –); and log2 fold change more than 3 have extreme changes (+ + + or – – –). The fraction of NB HMRs that intersected CGIs was colored in dark green and light green otherwise. Besides BT089, the HMRs from other methylomes, especially cell lines, are generally wider than NB. There were also a noticeable proportion of CGI-containing HMRs that became narrower in MCF7 and HCC1954.
(DOCX)

S2 Fig The DNA methylation and mRNA expression of *PDGFRB*, *VCAN*, and *SPDEF* in normal breast and MCF7 cells. Analysis of cloned amplified bisulfite-treated DNA containing upstream sequences of (A) *PDGFRB*, (B) *VCAN* and (C) *SPDEF* from normal breast and MCF7. Solid circles are methylated CpG sites and open circles indicate unmethylated CpG sites. (D) qRT-PCR expression levels of the three target genes was calibrated for each gene using 18S as housekeeping gene and normalized using the pool of normal breast replicates (∆∆Ct). Higher expression is equivalent to a smaller ∆∆Ct value. P-values are calculated using t-test.
(DOCX)

S3 Fig Examples of highly expressed genes that were hypermethylated. In HMEC and HCC1954, we identified four non-coding RNAs, namely (A) *U1*, (B) *SCARNA7* (C) *SCARNA9L* and (D) *SNORD71* that were both highly expressed and highly methylated at and around the genes in both cell lines. (E) We also found the highly expressed protein-coding gene *PPP2R2D* to be also completely methylated in normal breast (NB) and MCF7 cells.
(DOCX)

S4 Fig The DNA methylation and mRNA expression of *PPP2R2D* in normal breast and MCF7 cells. (A) Analysis of cloned amplified bisulfite-treated DNA containing *PPP2R2D* upstream sequences from normal breast and MCF7. Solid circles are methylated CpG sites and open circles indicate unmethylated CpG sites. (B) qRT-PCR expression levels of *PPP2R2D* and *ACTB* was calibrated using 18S as housekeeping gene and expressed as ∆Ct. Compared with the housekeeping gene *ACTB*, *PPP2R2D* is moderately expressed in both samples despite the presence of promoter methylation.
(DOCX)

S5 Fig Scatter plots of male (x-axis) and female (y-axis) DNA methylation levels of gene promoters located in the X chromosomes. Promoters whose methylation levels were comparable between male and female methylomes clustered along the diagonal line. Due to the effect of XCI, one copy of the female X chromosome is mostly inactivated and methylated, while the other copy remains active and hypomethylated at regulatory sites such as promoters. Promoters under the influence of XCI in females will show 50% methylation at CpG sites on the X chromosome while males will show substantially less methylation. Regions that showed male-female differences were marked with gray squares.
(DOCX)

S6 Fig Regulatory properties of the 24 clusters of reference HMRs. (A) Distribution of lengths of HMRs. (B) CpG density of HMRs expressed as the number of CpG sites per 100 bp of nucleotide sequence. Proportions of HMRs in each cluster that intersected (C) CGI, (D) RNA PolII binding sites from MCF7 and ENCODE. (E) ENCODE TFBS, (F) FANTOM5 TSS, (G) FANTOM5 enhancers, (H) ENCODE DNase I hypersensitive sites (HS), (I) MCF7 MNase hypersensitive sites, and (J) MCF7 HpaII hypersensitive sites.
(DOCX)

S7 Fig Association of HMRs with ENCODE histone modification data. The bar plots showed the proportion of HMRs that harbored high levels of (A) H3k4me1, (B) H3k4me2, (C) H3k4me3, (D) H3k27me3, (E) H3k27ac, and (F) H3k9ac. For each histone modification, the HMRs whose score is in the top 20% were considered as having high levels.
(DOCX)

S8 Fig Association of HMRs with MCF7 ChIP-chip data. The bar plots showed the proportion of HMRs that harbored high levels of (A) H3k4me1, (B) H3k4me3, (C) H3K4ac, (D) H3K9ac, (E) CTCF, (F) HP1a, (G) H3K27me3 and (H) H3K9me2. For each ChIP-chip data, the HMRs whose score is in the top 20% were considered as having high levels.
(DOCX)

S9 Fig Visualization of four enhancers/regulatory elements that exhibited tumor-specific hypomethylation and hypermethylation of the associated HMR. (A) Differential hypomethylation of the distal enhancer (C-5 HMR) of downstream *MYC* gene. (B) Differential hypermethylation of the internal enhancer (A-7 HMR) in the second intron of *NOTCH1*. (C) Differential hypomethylation of the DNA replication initiation site (A-8 HMR) located in the first intron of *DNMT1*. (D) Differential hypomethylation of the estrogen receptor binding sites (B-5 HMR) within intron 2 of SLC22A5. The regions of interest were boxed and highlighted in yellow. The DNA methylation levels of the seven methylomes were displayed as red-green tracks where red indicates methylation levels > 50% and green indicates methylation levels < 50%.
(DOCX)

S10 Fig Percentage of nine ChromHMM states that intersect the 24 HMR clusters. The HMEC and MCF7 chromatin data were colored in green and blue respectively. The HMR clusters that showed significant difference in the proportion of states between HMEC and MCF7 were marked with asterisk (z-test p-value < 1.E-50).
(DOCX)

S11 Fig PMDs are associated with CpG-poor and gene-poor genomic regions and lamin B1. The distribution of (A) CpG densities, (B) gene density and (C) average NKI LaminB1 score of PMD and non-PMD (nPMD) regions. The CpG and gene density was expressed as the number of CpG sites and number of genes per 100 bp of nucleotide sequence respectively. The medium values were provided above each boxplot.
(DOCX)

S12 Fig Fluorescence *in situ* hybridization (FISH) analysis revealed localization of McrBC-resistant hypomethylated DNA in the MCF7 nuclei. The Restriction enzyme McrBC will specifically cleaving methylated CpG sites, leaving unmethylated DNA intact. The FISH signals were found at the nuclear periphery with chromosomal DNA counterstained with DAPI.
(DOCX)

S13 Fig Common fragile sites are associated with hypomethylated breast tumor PMDs. (A) The proportion of 127 fragile sites overlapped predicted PMDs of the seven methylomes. More than 50% of the fragile sites in BT198 and breast cell lines were associated with PMDs. nPMD: sites that do not overlapped PMD. PMD: sites that contain PMDs. (B) The distribution of fragile sites DNA methylation. The methylation levels of PMD-containing fragile sites were significantly lower than those not having PMDs in BT126, BT198, HMEC, HCC1954 and MCF7 (t-test, p-value < 0.01).
(DOCX)

S1 Table Whole-genome bisulfite sequencing and mapping statistics of the seven breast methylomes. (XLSX)

S2 Table Characteristics of the hypomethylated regions (HMRs) identified in the seven breast methylomes. (XLSX)

S3 Table Differential expression analysis of tumor suppressor genes and transcription regulators that showed promoter HMR expansion or contraction. (XLSX)

S4 Table Function analysis of genes associated with A-1 and A-4 promoter HMRs. (XLSX)

S5 Table Function analysis of differentially expressed genes associated with A-3, A-6 and A-8 HMRs. (XLSX)

S6 Table Function analysis of differentially expressed genes associated with A-2, A-5 and A-7 HMRs. (XLSX)

S7 Table List of HMRs used for methylation analysis of the BRCA dataset. (XLSX)

S8 Table Function analysis of genes associated with A-6 promoter HMRs (limited to genes with sufficient CpG coverage on HumanMethylation450 BeadChip). (XLSX)

S9 Table List of genes located on the X chromosome that had hypomethylated promoters and were overexpressed in MCF7 and HCC1954. (XLSX)

S10 Table List of genes located on the X chromosome that had hypomethylated promoters and were overexpressed in the TCGA BRCA samples. (XLSX)

S11 Table Characteristics of the partially hypomethylated domains (PMDs) identified in the seven breast methylomes. (XLSX)

S12 Table Primer sequences used for nested PCR of bisulfite genomic DNA. (XLSX)

S13 Table The list of BRCA samples used. (XLSX)

## References

[1] Huang Y, Nayak S, Jankowitz R, Davidson NE, Oesterreich S. Epigenetics in breast cancer: what’s new? Breast Cancer Res. 2011;13(6):225 10.1186/bcr2925 22078060 PMC3326545

[2] Esteller M. Cancer epigenomics: DNA methylomes and histone-modification maps. Nature reviews Genetics. 2007;8(4):286–98. 17339880 10.1038/nrg2005

[3] Locke WJ, Clark SJ. Epigenome remodelling in breast cancer: insights from an early in vitro model of carcinogenesis. Breast Cancer Res. 2012;14(6):215 10.1186/bcr3237 23168266 PMC4053120

[4] Reik W. Stability and flexibility of epigenetic gene regulation in mammalian development. Nature. 2007;447(7143):425–32. 17522676 10.1038/nature05918

[5] Meissner A, Mikkelsen TS, Gu H, Wernig M, Hanna J, Sivachenko A, et al Genome-scale DNA methylation maps of pluripotent and differentiated cells. Nature. 2008;454(7205):766–70. 10.1038/nature07107 18600261 PMC2896277

[6] Ehrlich M. DNA hypomethylation in cancer cells. Epigenomics. 2009;1(2):239–59. 10.2217/epi.09.33 20495664 PMC2873040

[7] Park YJ, Claus R, Weichenhan D, Plass C. Genome-wide epigenetic modifications in cancer. Progress in drug research Fortschritte der Arzneimittelforschung Progres des recherches pharmaceutiques. 2011;67:25–49. 21141723 10.1007/978-3-7643-8989-5_2 PMC3066002

[8] Chaligne R, Heard E. X-chromosome inactivation in development and cancer. FEBS Lett. 2014;588(15):2514–22. 10.1016/j.febslet.2014.06.023 24937141

[9] Bird A. DNA methylation patterns and epigenetic memory. Genes Dev. 2002;16(1):6–21. 11782440 10.1101/gad.947102

[10] Weber M, Hellmann I, Stadler MB, Ramos L, Paabo S, Rebhan M, et al Distribution, silencing potential and evolutionary impact of promoter DNA methylation in the human genome. Nat Genet. 2007;39(4):457–66. 17334365 10.1038/ng1990

[11] Jones PA, Baylin SB. The epigenomics of cancer. Cell. 2007;128(4):683–92. 17320506 10.1016/j.cell.2007.01.029 PMC3894624

[12] Deaton AM, Bird A. CpG islands and the regulation of transcription. Genes Dev. 2011;25(10):1010–22. 10.1101/gad.2037511 21576262 PMC3093116

[13] Gupta A, Godwin AK, Vanderveer L, Lu A, Liu J. Hypomethylation of the synuclein gamma gene CpG island promotes its aberrant expression in breast carcinoma and ovarian carcinoma. Cancer Res. 2003;63(3):664–73. 12566312

[14] Futscher BW, O’Meara MM, Kim CJ, Rennels MA, Lu D, Gruman LM, et al Aberrant methylation of the maspin promoter is an early event in human breast cancer. Neoplasia. 2004;6(4):380–9. 15256060 10.1593/neo.04115 PMC1502109

[15] Wilson AS, Power BE, Molloy PL. DNA hypomethylation and human diseases. Biochim Biophys Acta. 2007;1775(1):138–62. 17045745 10.1016/j.bbcan.2006.08.007

[16] Roll JD, Rivenbark AG, Jones WD, Coleman WB. DNMT3b overexpression contributes to a hypermethylator phenotype in human breast cancer cell lines. Molecular cancer. 2008;7:15 10.1186/1476-4598-7-15 18221536 PMC2246151

[17] Han LL, Hou L, Zhou MJ, Ma ZL, Lin DL, Wu L, et al Aberrant NDRG1 methylation associated with its decreased expression and clinicopathological significance in breast cancer. J Biomed Sci. 2013;20:52 10.1186/1423-0127-20-52 23899187 PMC3751627

[18] Irizarry RA, Ladd-Acosta C, Wen B, Wu Z, Montano C, Onyango P, et al The human colon cancer methylome shows similar hypo- and hypermethylation at conserved tissue-specific CpG island shores. Nat Genet. 2009;41(2):178–86. 10.1038/ng.298 19151715 PMC2729128

[19] Han H, Cortez CC, Yang X, Nichols PW, Jones PA, Liang G. DNA methylation directly silences genes with non-CpG island promoters and establishes a nucleosome occupied promoter. Hum Mol Genet. 2011;20(22):4299–310. 10.1093/hmg/ddr356 21835883 PMC3196883

[20] Shenker N, Flanagan JM. Intragenic DNA methylation: implications of this epigenetic mechanism for cancer research. Br J Cancer. 2012;106(2):248–53. 10.1038/bjc.2011.550 22166804 PMC3261681

[21] Yu YP, Ding Y, Chen R, Liao SG, Ren BG, Michalopoulos A, et al Whole-genome methylation sequencing reveals distinct impact of differential methylations on gene transcription in prostate cancer. Am J Pathol. 2013;183(6):1960–70. 10.1016/j.ajpath.2013.08.018 24113458 PMC5745540

[22] Reyngold M, Turcan S, Giri D, Kannan K, Walsh LA, Viale A, et al Remodeling of the methylation landscape in breast cancer metastasis. PLoS One. 2014;9(8):e103896 10.1371/journal.pone.0103896 25083786 PMC4118917

[23] Illingworth R, Kerr A, Desousa D, Jorgensen H, Ellis P, Stalker J, et al A novel CpG island set identifies tissue-specific methylation at developmental gene loci. PLoS Biol. 2008;6(1):e22 10.1371/journal.pbio.0060022 18232738 PMC2214817

[24] Davies MN, Volta M, Pidsley R, Lunnon K, Dixit A, Lovestone S, et al Functional annotation of the human brain methylome identifies tissue-specific epigenetic variation across brain and blood. Genome biology. 2012;13(6):R43 10.1186/gb-2012-13-6-r43 22703893 PMC3446315

[25] Schlesinger F, Smith AD, Gingeras TR, Hannon GJ, Hodges E. De novo DNA demethylation and noncoding transcription define active intergenic regulatory elements. Genome Res. 2013;23(10):1601–14. 10.1101/gr.157271.113 23811145 PMC3787258

[26] Stirzaker C, Taberlay PC, Statham AL, Clark SJ. Mining cancer methylomes: prospects and challenges. Trends Genet. 2014;30(2):75–84. 10.1016/j.tig.2013.11.004 24368016

[27] Li L, Lee KM, Han W, Choi JY, Lee JY, Kang GH, et al Estrogen and progesterone receptor status affect genome-wide DNA methylation profile in breast cancer. Hum Mol Genet. 2010;19(21):4273–7. 10.1093/hmg/ddq351 20724461

[28] Fackler MJ, Umbricht CB, Williams D, Argani P, Cruz LA, Merino VF, et al Genome-wide methylation analysis identifies genes specific to breast cancer hormone receptor status and risk of recurrence. Cancer Res. 2011;71(19):6195–207. 10.1158/0008-5472.CAN-11-1630 21825015 PMC3308629

[29] Faryna M, Konermann C, Aulmann S, Bermejo JL, Brugger M, Diederichs S, et al Genome-wide methylation screen in low-grade breast cancer identifies novel epigenetically altered genes as potential biomarkers for tumor diagnosis. FASEB journal: official publication of the Federation of American Societies for Experimental Biology. 2012;26(12):4937–50. 10.1096/fj.12-209502 22930747

[30] Avraham A, Cho SS, Uhlmann R, Polak ML, Sandbank J, Karni T, et al Tissue specific DNA methylation in normal human breast epithelium and in breast cancer. PLoS One. 2014;9(3):e91805 10.1371/journal.pone.0091805 24651077 PMC3961270

[31] Hill VK, Ricketts C, Bieche I, Vacher S, Gentle D, Lewis C, et al Genome-wide DNA methylation profiling of CpG islands in breast cancer identifies novel genes associated with tumorigenicity. Cancer Res. 2011;71(8):2988–99. 10.1158/0008-5472.CAN-10-4026 21363912

[32] Lister R, Pelizzola M, Dowen RH, Hawkins RD, Hon G, Tonti-Filippini J, et al Human DNA methylomes at base resolution show widespread epigenomic differences. Nature. 2009;462(7271):315–22. 10.1038/nature08514 19829295 PMC2857523

[33] Li Y, Zhu J, Tian G, Li N, Li Q, Ye M, et al The DNA methylome of human peripheral blood mononuclear cells. PLoS Biol. 2010;8(11):e1000533 10.1371/journal.pbio.1000533 21085693 PMC2976721

[34] Laurent L, Wong E, Li G, Huynh T, Tsirigos A, Ong CT, et al Dynamic changes in the human methylome during differentiation. Genome Res. 2010;20(3):320–31. 10.1101/gr.101907.109 20133333 PMC2840979

[35] Molaro A, Hodges E, Fang F, Song Q, McCombie WR, Hannon GJ, et al Sperm methylation profiles reveal features of epigenetic inheritance and evolution in primates. Cell. 2011;146(6):1029–41. 10.1016/j.cell.2011.08.016 21925323 PMC3205962

[36] Lister R, Pelizzola M, Kida YS, Hawkins RD, Nery JR, Hon G, et al Hotspots of aberrant epigenomic reprogramming in human induced pluripotent stem cells. Nature. 2011;471(7336):68–73. 10.1038/nature09798 21289626 PMC3100360

[37] Hodges E, Molaro A, Dos Santos CO, Thekkat P, Song Q, Uren PJ, et al Directional DNA methylation changes and complex intermediate states accompany lineage specificity in the adult hematopoietic compartment. Mol Cell. 2011;44(1):17–28. 10.1016/j.molcel.2011.08.026 21924933 PMC3412369

[38] Hon GC, Hawkins RD, Caballero OL, Lo C, Lister R, Pelizzola M, et al Global DNA hypomethylation coupled to repressive chromatin domain formation and gene silencing in breast cancer. Genome Res. 2012;22(2):246–58. 10.1101/gr.125872.111 22156296 PMC3266032

[39] Heyn H, Li N, Ferreira HJ, Moran S, Pisano DG, Gomez A, et al Distinct DNA methylomes of newborns and centenarians. Proceedings of the National Academy of Sciences of the United States of America. 2012;109(26):10522–7. 10.1073/pnas.1120658109 22689993 PMC3387108

[40] Berman BP, Weisenberger DJ, Aman JF, Hinoue T, Ramjan Z, Liu Y, et al Regions of focal DNA hypermethylation and long-range hypomethylation in colorectal cancer coincide with nuclear lamina-associated domains. Nat Genet. 2012;44(1):40–6. 10.1038/ng.969 PMC4309644 22120008

[41] Ziller MJ, Gu H, Muller F, Donaghey J, Tsai LT, Kohlbacher O, et al Charting a dynamic DNA methylation landscape of the human genome. Nature. 2013;500(7463):477–81. 10.1038/nature12433 23925113 PMC3821869

[42] Sproul D, Meehan RR. Genomic insights into cancer-associated aberrant CpG island hypermethylation. Briefings in functional genomics. 2013;12(3):174–90. 10.1093/bfgp/els063 23341493 PMC3662888

[43] Cohen J. Statistical Power Analysis for the Behavioral Sciences: L. Erlbaum Associates; 1988.

[44] Penny GD, Kay GF, Sheardown SA, Rastan S, Brockdorff N. Requirement for Xist in X chromosome inactivation. Nature. 1996;379(6561):131–7. 8538762 10.1038/379131a0

[45] Wutz A, Jaenisch R. A shift from reversible to irreversible X inactivation is triggered during ES cell differentiation. Mol Cell. 2000;5(4):695–705. 10882105 10.1016/s1097-2765(00)80248-8

[46] Zhang Y, Castillo-Morales A, Jiang M, Zhu Y, Hu L, Urrutia AO, et al Genes that escape X-inactivation in humans have high intraspecific variability in expression, are associated with mental impairment but are not slow evolving. Mol Biol Evol. 2013;30(12):2588–601. 10.1093/molbev/mst148 24023392 PMC3840307

[47] Wang C, Mayer JA, Mazumdar A, Fertuck K, Kim H, Brown M, et al Estrogen induces c-myc gene expression via an upstream enhancer activated by the estrogen receptor and the AP-1 transcription factor. Mol Endocrinol. 2011;25(9):1527–38. 10.1210/me.2011-1037 21835891 PMC3165912

[48] Chalamalasetty RB, Dunty WC Jr., Biris KK, Ajima R, Iacovino M, Beisaw A, et al The Wnt3a/beta-catenin target gene Mesogenin1 controls the segmentation clock by activating a Notch signalling program. Nature communications. 2011;2:390 10.1038/ncomms1381 21750544 PMC3622708

[49] Araujo FD, Knox JD, Ramchandani S, Pelletier R, Bigey P, Price G, et al Identification of initiation sites for DNA replication in the human dnmt1 (DNA-methyltransferase) locus. J Biol Chem. 1999;274(14):9335–41. 10092611 10.1074/jbc.274.14.9335

[50] Wang C, Uray IP, Mazumdar A, Mayer JA, Brown PH. SLC22A5/OCTN2 expression in breast cancer is induced by estrogen via a novel intronic estrogen-response element (ERE). Breast cancer research and treatment. 2012;134(1):101–15. 10.1007/s10549-011-1925-0 22212555 PMC3416040

[51] Taberlay PC, Statham AL, Kelly TK, Clark SJ, Jones PA. Reconfiguration of nucleosome depleted regions at distal regulatory elements accompanies DNA methylation of enhancers and insulators in cancer. Genome Res. 2014 10.1101/gr.174730.114 24916973 PMC4158760

[52] Hovestadt V, Jones DT, Picelli S, Wang W, Kool M, Northcott PA, et al Decoding the regulatory landscape of medulloblastoma using DNA methylation sequencing. Nature. 2014;510(7506):537–41. 10.1038/nature13268 24847876

[53] Marzese DM, Scolyer RA, Huynh JL, Huang SK, Hirose H, Chong KK, et al Epigenome-wide DNA methylation landscape of melanoma progression to brain metastasis reveals aberrations on homeobox D cluster associated with prognosis. Hum Mol Genet. 2014;23(1):226–38. 10.1093/hmg/ddt420 24014427 PMC3857956

[54] Liu S, Cong Y, Wang D, Sun Y, Deng L, Liu Y, et al Breast Cancer Stem Cells Transition between Epithelial and Mesenchymal States Reflective of their Normal Counterparts. Stem Cell Reports. 2014;2(1):78–91. 10.1016/j.stemcr.2013.11.009 24511467 PMC3916760

[55] Wan L, Pantel K, Kang Y. Tumor metastasis: moving new biological insights into the clinic. Nat Med. 2013;19(11):1450–64. 10.1038/nm.3391 24202397

[56] Thakur A, Rahman KW, Wu J, Bollig A, Biliran H, Lin X, et al Aberrant expression of X-linked genes RbAp46, Rsk4, and Cldn2 in breast cancer. Mol Cancer Res. 2007;5(2):171–81. 17314274 10.1158/1541-7786.MCR-06-0071

[57] Anguera MC, Sadreyev R, Zhang Z, Szanto A, Payer B, Sheridan SD, et al Molecular signatures of human induced pluripotent stem cells highlight sex differences and cancer genes. Cell Stem Cell. 2012;11(1):75–90. 10.1016/j.stem.2012.03.008 22770242 PMC3587778

[58] Jaenisch R, Bird A. Epigenetic regulation of gene expression: how the genome integrates intrinsic and environmental signals. Nat Genet. 2003;33 Suppl:245–54. 12610534 10.1038/ng1089

[59] Lung HL, Bangarusamy DK, Xie D, Cheung AK, Cheng Y, Kumaran MK, et al THY1 is a candidate tumour suppressor gene with decreased expression in metastatic nasopharyngeal carcinoma. Oncogene. 2005;24(43):6525–32. 16007174 10.1038/sj.onc.1208812

[60] Yamashita K, Katoh H, Watanabe M. The homeobox only protein homeobox (HOPX) and colorectal cancer. International journal of molecular sciences. 2013;14(12):23231–43. 10.3390/ijms141223231 24287901 PMC3876040

[61] Cedar H, Bergman Y. Linking DNA methylation and histone modification: patterns and paradigms. Nat Rev Genet. 2009;10(5):295–304. 10.1038/nrg2540 19308066

[62] Tsai Y-C, Chiao C-H, Chang IY-F, Chen D-T, Liu T-T, Hua K, et al Common Altered Epigenomic Domains in Cancer Cells: Characterization and Subtle Variations. Cancers. 2011;3(2):1996–2013. 10.3390/cancers3021996 24212793 PMC3757401

[63] Shann YJ, Cheng C, Chiao CH, Chen DT, Li PH, Hsu MT. Genome-wide mapping and characterization of hypomethylated sites in human tissues and breast cancer cell lines. Genome Res. 2008;18(5):791–801. 10.1101/gr.070961.107 18256232 PMC2336806

[64] Kuchiba A, Iwasaki M, Ono H, Kasuga Y, Yokoyama S, Onuma H, et al Global methylation levels in peripheral blood leukocyte DNA by LUMA and breast cancer: a case-control study in Japanese women. Br J Cancer. 2014;110(11):2765–71. 10.1038/bjc.2014.223 24786600 PMC4037832

[65] Song Q, Decato B, Hong EE, Zhou M, Fang F, Qu J, et al A reference methylome database and analysis pipeline to facilitate integrative and comparative epigenomics. PLoS One. 2013;8(12):e81148 10.1371/journal.pone.0081148 24324667 PMC3855694

[66] Dobin A, Davis CA, Schlesinger F, Drenkow J, Zaleski C, Jha S, et al STAR: ultrafast universal RNA-seq aligner. Bioinformatics. 2013;29(1):15–21. 10.1093/bioinformatics/bts635 23104886 PMC3530905

[67] Robinson MD, McCarthy DJ, Smyth GK. edgeR: a Bioconductor package for differential expression analysis of digital gene expression data. Bioinformatics. 2010;26(1):139–40. 10.1093/bioinformatics/btp616 19910308 PMC2796818

[68] Liao Y, Smyth GK, Shi W. featureCounts: an efficient general purpose program for assigning sequence reads to genomic features. Bioinformatics. 2014;30(7):923–30. 10.1093/bioinformatics/btt656 24227677

[69] Langmead B, Salzberg SL. Fast gapped-read alignment with Bowtie 2. Nat Methods. 2012;9(4):357–9. 10.1038/nmeth.1923 22388286 PMC3322381

[70] Kundaje A, Jung YL, Kharchenko P, Wold B, Sidow A, Batzoglou S, et al. Assessment of ChIP-seq data quality using cross-correlation analysis. Submitted.

[71] Zhang Y, Liu T, Meyer CA, Eeckhoute J, Johnson DS, Bernstein BE, et al Model-based analysis of ChIP-Seq (MACS). Genome biology. 2008;9(9):R137 10.1186/gb-2008-9-9-r137 18798982 PMC2592715

[72] Kundaje A, Li Q, Brown JB, Rozowsky J, Harmanci A, Wilder S, et al. Reproducibility measures for automatic threshold selection and quality control in ChIP-seq datasets. Submitted.

[73] Li H. Aligning sequence reads, clone sequences and assembly contigs with BWA-MEM. ArXiv e-prints [Internet]. 2013 March 1, 2013; 1303:[3997 p.]. Available from: http://adsabs.harvard.edu/abs/2013arXiv1303.3997L.

[74] Gyorffy B, Lanczky A, Eklund AC, Denkert C, Budczies J, Li Q, et al An online survival analysis tool to rapidly assess the effect of 22,277 genes on breast cancer prognosis using microarray data of 1,809 patients. Breast cancer research and treatment. 2010;123(3):725–31. 10.1007/s10549-009-0674-9 20020197

[75] Zhao H, Sun Z, Wang J, Huang H, Kocher JP, Wang L. CrossMap: a versatile tool for coordinate conversion between genome assemblies. Bioinformatics. 2014;30(7):1006–7. 10.1093/bioinformatics/btt730 24351709 PMC3967108

