# Supplementary material for: Hierarchical Clustering of Breast Cancer Methylomes Revealed Differentially Methylated and Expressed Breast Cancer Genes
Source: PLoS One. 2015 Feb 23;10(2):e0118453. doi: 10.1371/journal.pone.0118453 (PMC4338251; doi:10.1371/journal.pone.0118453)
Supplement: S1 Fig — (A) We compare the size of normal breast (NB) HMRs and the intersected CGI and showed HMR are generally wider than CGI. This information implied CGI shores, regions directly flanking CGI, are also hypomethylated. (B) The HMR sequences that directly overlapped CGIs and CGI shores were both associated with high number of TFBS compared with randomly selected regions from human genome. (C) The HMRs identified in NB were compared with those in primary breast tumors (BT089, BT126 and BT198) and breast cell lines (HMEC, MCF7 and HCC1954). In the x-axis, the plus (+) symbol denotes expansion and minus (–) symbol denotes contraction of the NB HMRs. HMRs that had log2 fold change between 0.19 ~ 1 have small changes in widths (+ or –); log2 fold change between 1 ~ 3 have large changes (+ + or – –); and log2 fold change more than 3 have extreme changes (+ + + or – – –). The fraction of NB HMRs that intersected CGIs was colored in dark green and light green otherwise. Besides BT089, the HMRs from other methylomes, especially cell lines, are generally wider than NB. There were also a noticeable proportion of CGI-containing HMRs that became narrower in MCF7 and HCC1954. (DOCX) [file pone.0118453.s001.docx]

**Figure S1. HMR properties.** (A) We compare the size of normal breast (NB) HMRs and the intersected CGI and showed HMR are generally wider than CGI. This information implied CGI shores, regions directly flanking CGI, are also hypomethylated. (B) The HMR sequences that directly overlapped CGIs and CGI shores were both associated with high number of TFBS compared with randomly selected regions from human genome. (C) The HMRs identified in NB were compared with those in primary breast tumors (BT089, BT126 and BT198) and breast cell lines (HMEC, MCF7 and HCC1954). In the x-axis, the plus (+) symbol denotes expansion and minus (–) symbol denotes contraction of the NB HMRs. HMRs that had log2 fold change between 0.19 ~ 1 have small changes in widths (+ or –); log2 fold change between 1 ~ 3 have large changes (+ + or – –); and log2 fold change more than 3 have extreme changes (+ + + or – – –). The fraction of NB HMRs that intersected CGIs was colored in dark green and light green otherwise. Besides BT089, the HMRs from other methylomes, especially cell lines, are generally wider than NB. There were also a noticeable proportion of CGI-containing HMRs that became narrower in MCF7 and HCC1954.
