# Supplementary material for: Hierarchical Clustering of Breast Cancer Methylomes Revealed Differentially Methylated and Expressed Breast Cancer Genes
Source: PLoS One. 2015 Feb 23;10(2):e0118453. doi: 10.1371/journal.pone.0118453 (PMC4338251; doi:10.1371/journal.pone.0118453)
Supplement: S2 Fig — Analysis of cloned amplified bisulfite-treated DNA containing upstream sequences of (A) PDGFRB, (B) VCAN and (C) SPDEF from normal breast and MCF7. Solid circles are methylated CpG sites and open circles indicate unmethylated CpG sites. (D) qRT-PCR expression levels of the three target genes was calibrated for each gene using 18S as housekeeping gene and normalized using the pool of normal breast replicates (∆∆Ct). Higher expression is equivalent to a smaller ∆∆Ct value. P-values are calculated using t-test. (DOCX) [file pone.0118453.s002.docx]

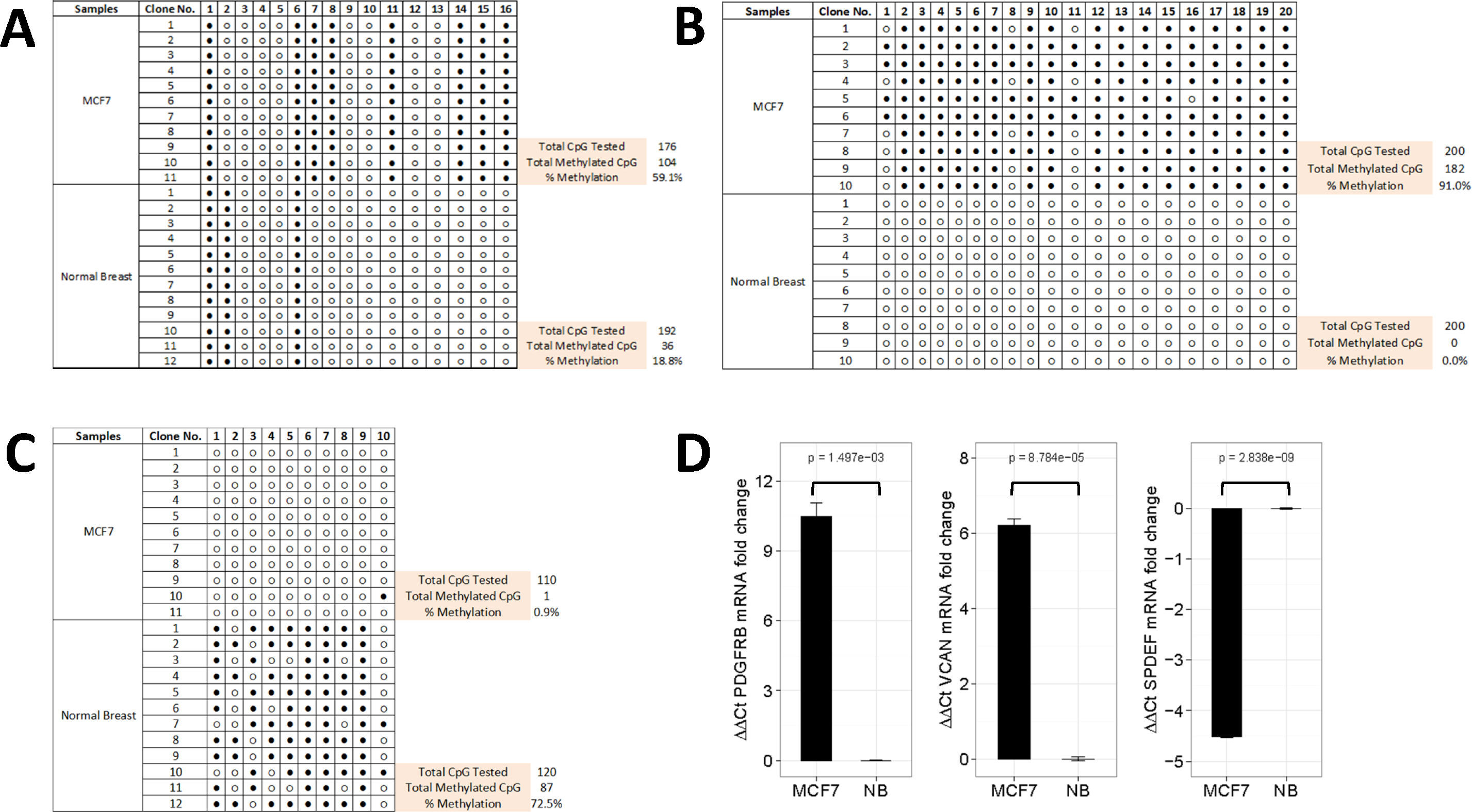


**Figure S2. The DNA methylation and mRNA expression of *PDGFRB*, *VCAN*, and *SPDEF* in normal breast and MCF7 cells.** Analysis of cloned amplified bisulfite-treated DNA containing upstream sequences of (A) *PDGFRB*, (B) *VCAN* and (C) *SPDEF* from normal breast and MCF7. Solid circles are methylated CpG sites and open circles indicate unmethylated CpG sites. (D) qRT-PCR expression levels of the three target genes was calibrated for each gene using 18S as housekeeping gene and normalized using the pool of normal breast replicates (∆∆Ct). Higher expression is equivalent to a smaller ∆∆Ct value. P-values are calculated using t-test.
