# Supplementary material for: Hierarchical Clustering of Breast Cancer Methylomes Revealed Differentially Methylated and Expressed Breast Cancer Genes
Source: PLoS One. 2015 Feb 23;10(2):e0118453. doi: 10.1371/journal.pone.0118453 (PMC4338251; doi:10.1371/journal.pone.0118453)
Supplement: S3 Fig — In HMEC and HCC1954, we identified four non-coding RNAs, namely (A) U1, (B) SCARNA7 (C) SCARNA9L and (D) SNORD71 that were both highly expressed and highly methylated at and around the genes in both cell lines. (E) We also found the highly expressed protein-coding gene PPP2R2D to be also completely methylated in normal breast (NB) and MCF7 cells. (DOCX) [file pone.0118453.s003.docx]

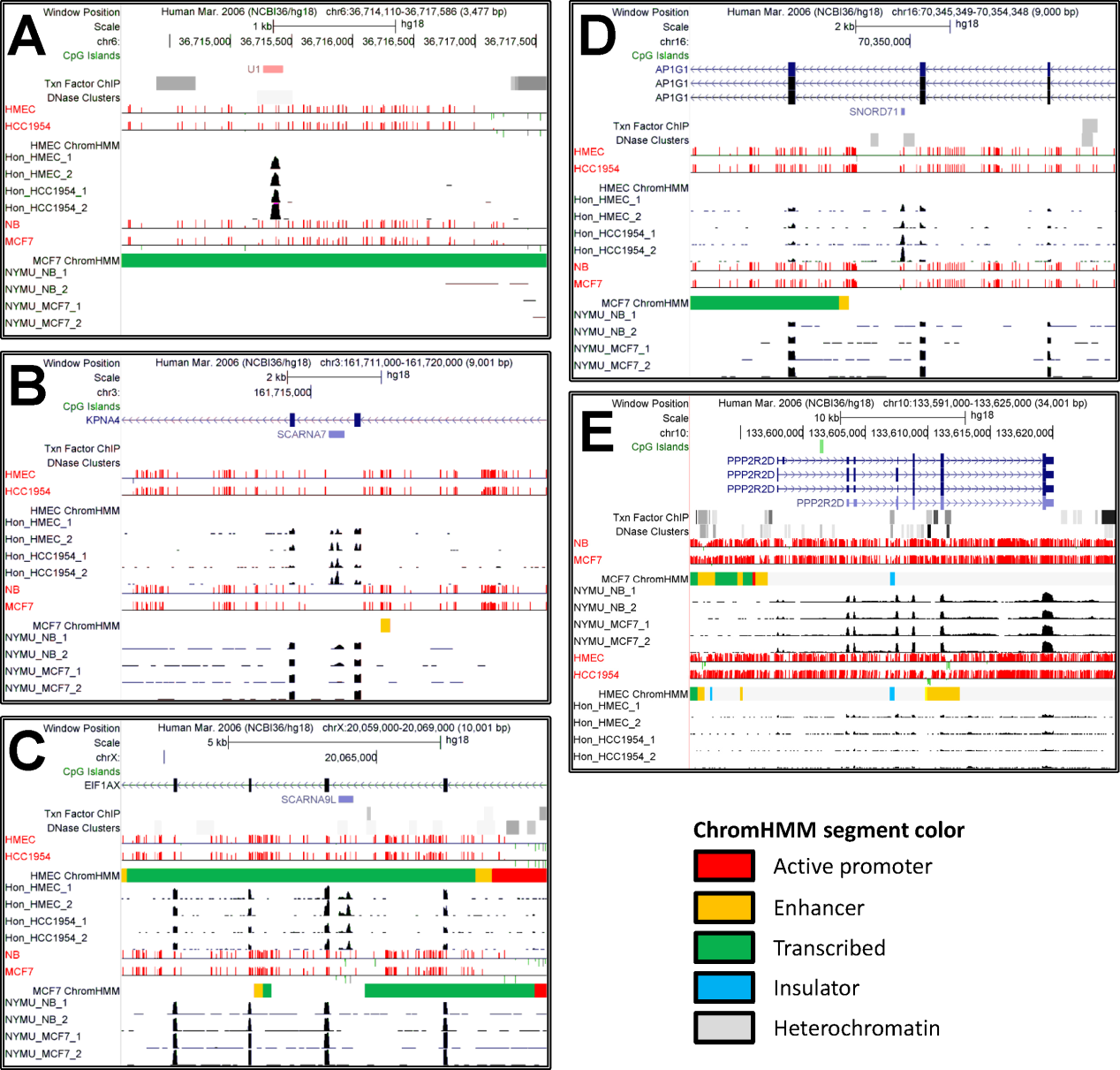


**Figure S3. Examples of highly expressed genes that were hypermethylated.** In HMEC and HCC1954, we identified four non-coding RNAs, namely (A) *U1*, (B) *SCARNA7* (C) *SCARNA9L* and (D) *SNORD71* that were both highly expressed and highly methylated at and around the genes in both cell lines. (E) We also found the highly expressed protein-coding gene *PPP2R2D* to be also completely methylated in normal breast (NB) and MCF7 cells.
