# Supplementary material for: Hierarchical Clustering of Breast Cancer Methylomes Revealed Differentially Methylated and Expressed Breast Cancer Genes
Source: PLoS One. 2015 Feb 23;10(2):e0118453. doi: 10.1371/journal.pone.0118453 (PMC4338251; doi:10.1371/journal.pone.0118453)
Supplement: S4 Fig — (A) Analysis of cloned amplified bisulfite-treated DNA containing PPP2R2D upstream sequences from normal breast and MCF7. Solid circles are methylated CpG sites and open circles indicate unmethylated CpG sites. (B) qRT-PCR expression levels of PPP2R2D and ACTB was calibrated using 18S as housekeeping gene and expressed as ∆Ct. Compared with the housekeeping gene ACTB, PPP2R2D is moderately expressed in both samples despite the presence of promoter methylation. (DOCX) [file pone.0118453.s004.docx]

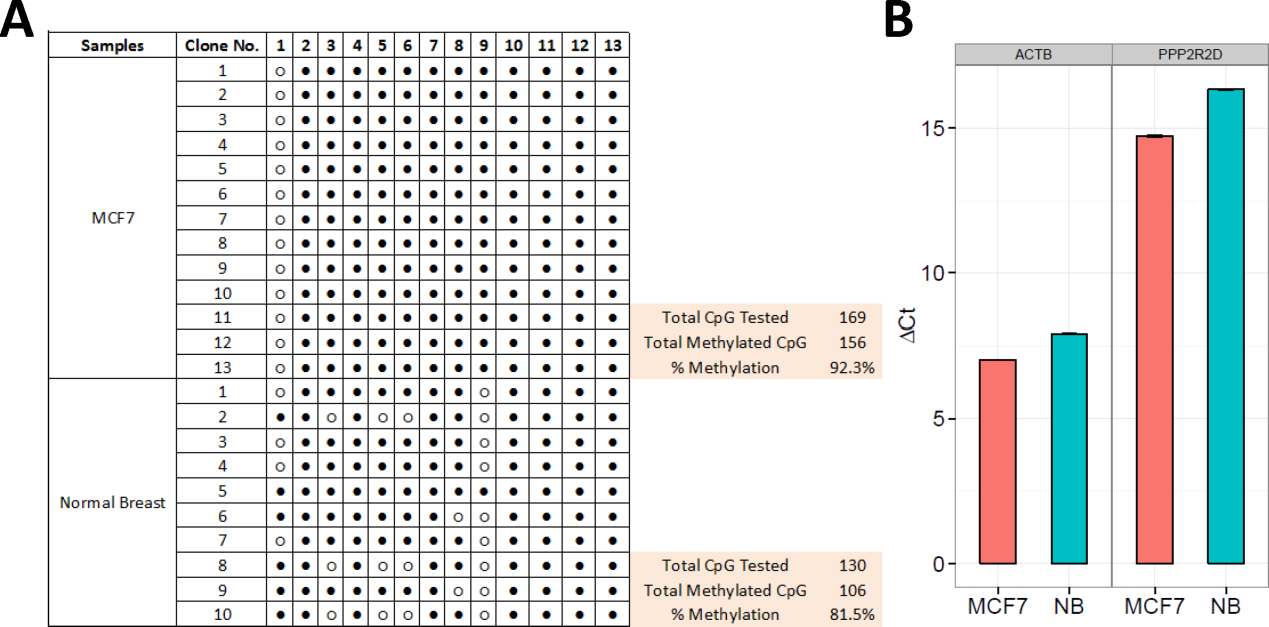


**Figure S4. The DNA methylation and mRNA expression of *PPP2R2D* in normal breast and MCF7 cells.** (A) Analysis of cloned amplified bisulfite-treated DNA containing *PPP2R2D* upstream sequences from normal breast and MCF7. Solid circles are methylated CpG sites and open circles indicate unmethylated CpG sites. (B) qRT-PCR expression levels of *PPP2R2D* and *ACTB* was calibrated using 18S as housekeeping gene and expressed as ∆Ct. Compared with the housekeeping gene *ACTB*, *PPP2R2D* is moderately expressed in both samples despite the presence of promoter methylation.
