# Supplementary material for: Hierarchical Clustering of Breast Cancer Methylomes Revealed Differentially Methylated and Expressed Breast Cancer Genes
Source: PLoS One. 2015 Feb 23;10(2):e0118453. doi: 10.1371/journal.pone.0118453 (PMC4338251; doi:10.1371/journal.pone.0118453)
Supplement: S5 Fig — Promoters whose methylation levels were comparable between male and female methylomes clustered along the diagonal line. Due to the effect of XCI, one copy of the female X chromosome is mostly inactivated and methylated, while the other copy remains active and hypomethylated at regulatory sites such as promoters. Promoters under the influence of XCI in females will show 50% methylation at CpG sites on the X chromosome while males will show substantially less methylation. Regions that showed male-female differences were marked with gray squares. (DOCX) [file pone.0118453.s005.docx]

**Figure S5. Scatter plots of male (x-axis) and female (y-axis) DNA methylation levels of gene promoters located in the X chromosomes.** Promoters whose methylation levels were comparable between male and female methylomes clustered along the diagonal line. Due to the effect of XCI, one copy of the female X chromosome is mostly inactivated and methylated, while the other copy remains active and hypomethylated at regulatory sites such as promoters. Promoters under the influence of XCI in females will show 50% methylation at CpG sites on the X chromosome while males will show substantially less methylation. Regions that showed male-female differences were marked with gray squares.
