# Supplementary material for: Hierarchical Clustering of Breast Cancer Methylomes Revealed Differentially Methylated and Expressed Breast Cancer Genes
Source: PLoS One. 2015 Feb 23;10(2):e0118453. doi: 10.1371/journal.pone.0118453 (PMC4338251; doi:10.1371/journal.pone.0118453)
Supplement: S6 Fig — (A) Distribution of lengths of HMRs. (B) CpG density of HMRs expressed as the number of CpG sites per 100 bp of nucleotide sequence. Proportions of HMRs in each cluster that intersected (C) CGI, (D) RNA PolII binding sites from MCF7 and ENCODE. (E) ENCODE TFBS, (F) FANTOM5 TSS, (G) FANTOM5 enhancers, (H) ENCODE DNase I hypersensitive sites (HS), (I) MCF7 MNase hypersensitive sites, and (J) MCF7 HpaII hypersensitive sites. (DOCX) [file pone.0118453.s006.docx]

**Figure S6. Regulatory properties of the 24 clusters of reference HMRs.** (A) Distribution of lengths of HMRs. (B) CpG density of HMRs expressed as the number of CpG sites per 100 bp of nucleotide sequence. Proportions of HMRs in each cluster that intersected (C) CGI, (D) RNA PolII binding sites from MCF7 and ENCODE. (E) ENCODE TFBS, (F) FANTOM5 TSS, (G) FANTOM5 enhancers, (H) ENCODE DNase I hypersensitive sites (HS), (I) MCF7 MNase hypersensitive sites, and (J) MCF7 HpaII hypersensitive sites.
