# Supplementary material for: Hierarchical Clustering of Breast Cancer Methylomes Revealed Differentially Methylated and Expressed Breast Cancer Genes
Source: PLoS One. 2015 Feb 23;10(2):e0118453. doi: 10.1371/journal.pone.0118453 (PMC4338251; doi:10.1371/journal.pone.0118453)
Supplement: S7 Fig — The bar plots showed the proportion of HMRs that harbored high levels of (A) H3k4me1, (B) H3k4me2, (C) H3k4me3, (D) H3k27me3, (E) H3k27ac, and (F) H3k9ac. For each histone modification, the HMRs whose score is in the top 20% were considered as having high levels. (DOCX) [file pone.0118453.s007.docx]

**Figure S7. Association of HMRs with ENCODE histone modification data.** The bar plots showed the proportion of HMRs that harbored high levels of (A) H3k4me1, (B) H3k4me2, (C) H3k4me3, (D) H3k27me3, (E) H3k27ac, and (F) H3k9ac. For each histone modification, the HMRs whose score is in the top 20% were considered as having high levels.
