# Supplementary material for: Hierarchical Clustering of Breast Cancer Methylomes Revealed Differentially Methylated and Expressed Breast Cancer Genes
Source: PLoS One. 2015 Feb 23;10(2):e0118453. doi: 10.1371/journal.pone.0118453 (PMC4338251; doi:10.1371/journal.pone.0118453)
Supplement: S8 Fig — The bar plots showed the proportion of HMRs that harbored high levels of (A) H3k4me1, (B) H3k4me3, (C) H3K4ac, (D) H3K9ac, (E) CTCF, (F) HP1a, (G) H3K27me3 and (H) H3K9me2. For each ChIP-chip data, the HMRs whose score is in the top 20% were considered as having high levels. (DOCX) [file pone.0118453.s008.docx]

**Figure S8. Association of HMRs with MCF7 ChIP-chip data.** The bar plots showed the proportion of HMRs that harbored high levels of (A) H3k4me1, (B) H3k4me3, (C) H3K4ac, (D) H3K9ac, (E) CTCF, (F) HP1a, (G) H3K27me3 and (H) H3K9me2. For each ChIP-chip data, the HMRs whose score is in the top 20% were considered as having high levels.
