# Supplementary material for: Hierarchical Clustering of Breast Cancer Methylomes Revealed Differentially Methylated and Expressed Breast Cancer Genes
Source: PLoS One. 2015 Feb 23;10(2):e0118453. doi: 10.1371/journal.pone.0118453 (PMC4338251; doi:10.1371/journal.pone.0118453)
Supplement: S9 Fig — (A) Differential hypomethylation of the distal enhancer (C-5 HMR) of downstream MYC gene. (B) Differential hypermethylation of the internal enhancer (A-7 HMR) in the second intron of NOTCH1. (C) Differential hypomethylation of the DNA replication initiation site (A-8 HMR) located in the first intron of DNMT1. (D) Differential hypomethylation of the estrogen receptor binding sites (B-5 HMR) within intron 2 of SLC22A5. The regions of interest were boxed and highlighted in yellow. The DNA methylation levels of the seven methylomes were displayed as red-green tracks where red indicates methylation levels > 50% and green indicates methylation levels < 50%. (DOCX) [file pone.0118453.s009.docx]

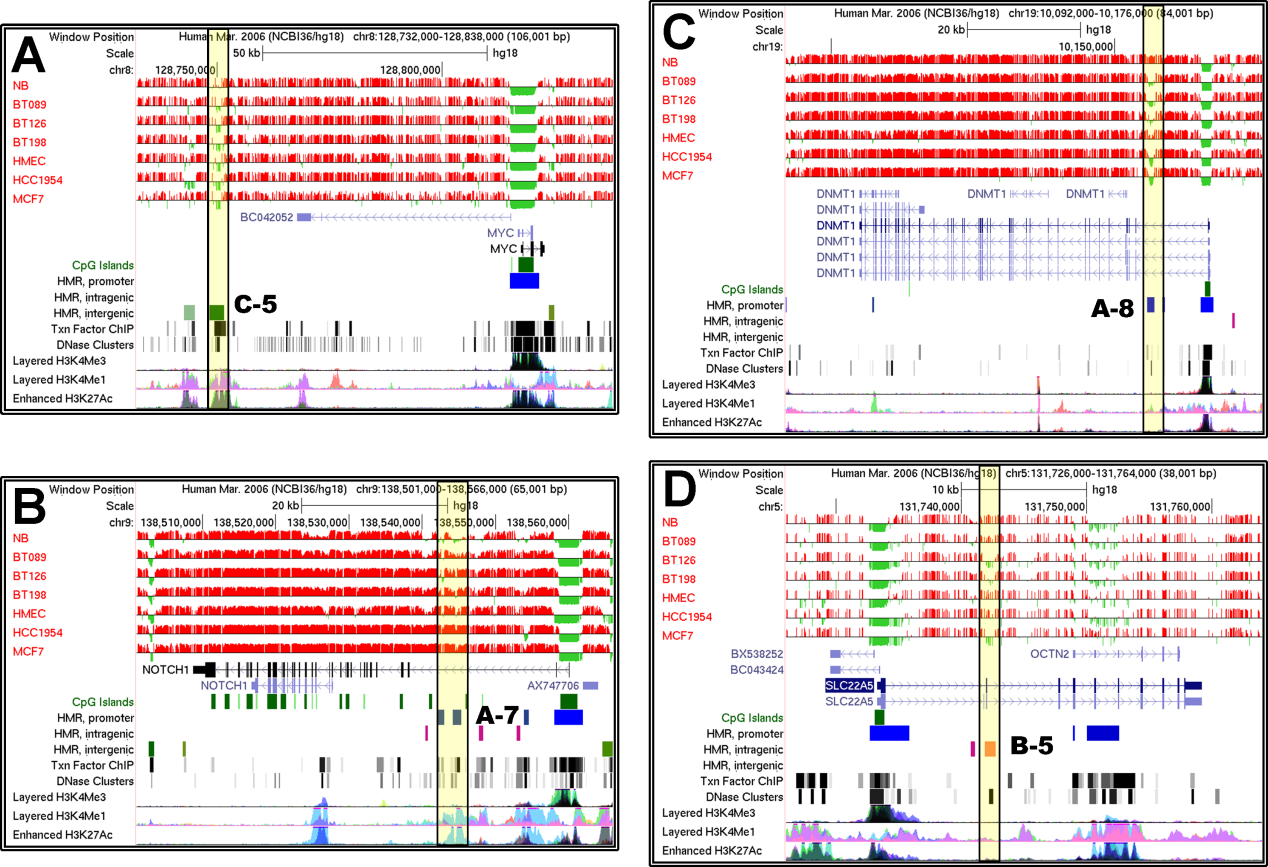


**Figure S9. Visualization of four enhancers/regulatory elements that exhibited tumor-specific hypomethylation and hypermethylation of the associated HMR.** (A) Differential hypomethylation of the distal enhancer (C-5 HMR) of downstream *MYC* gene. (B) Differential hypermethylation of the internal enhancer (A-7 HMR) in the second intron of *NOTCH1*. (C) Differential hypomethylation of the DNA replication initiation site (A-8 HMR) located in the first intron of *DNMT1*. (D) Differential hypomethylation of the estrogen receptor binding sites (B-5 HMR) within intron 2 of SLC22A5. The regions of interest were boxed and highlighted in yellow. The DNA methylation levels of the seven methylomes were displayed as red-green tracks where red indicates methylation levels > 50% and green indicates methylation levels < 50%.
