# Supplementary material for: Hierarchical Clustering of Breast Cancer Methylomes Revealed Differentially Methylated and Expressed Breast Cancer Genes
Source: PLoS One. 2015 Feb 23;10(2):e0118453. doi: 10.1371/journal.pone.0118453 (PMC4338251; doi:10.1371/journal.pone.0118453)
Supplement: S10 Fig — The HMEC and MCF7 chromatin data were colored in green and blue respectively. The HMR clusters that showed significant difference in the proportion of states between HMEC and MCF7 were marked with asterisk (z-test p-value < 1.E-50). (DOCX) [file pone.0118453.s010.docx]

**Figure S10. Percentage of nine ChromHMM states that intersect the 24 HMR clusters.** The HMEC and MCF7 chromatin data were colored in green and blue respectively. The HMR clusters that showed significant difference in the proportion of states between HMEC and MCF7 were marked with asterisk (z-test p-value < 1.E-50).
