# Supplementary material for: Hierarchical Clustering of Breast Cancer Methylomes Revealed Differentially Methylated and Expressed Breast Cancer Genes
Source: PLoS One. 2015 Feb 23;10(2):e0118453. doi: 10.1371/journal.pone.0118453 (PMC4338251; doi:10.1371/journal.pone.0118453)
Supplement: S11 Fig — The distribution of (A) CpG densities, (B) gene density and (C) average NKI LaminB1 score of PMD and non-PMD (nPMD) regions. The CpG and gene density was expressed as the number of CpG sites and number of genes per 100 bp of nucleotide sequence respectively. The medium values were provided above each boxplot. (DOCX) [file pone.0118453.s011.docx]

**Figure S11. PMDs are associated with CpG-poor and gene-poor genomic regions and lamin B1.** The distribution of (A) CpG densities, (B) gene density and (C) average NKI LaminB1 score of PMD and non-PMD (nPMD) regions. The CpG and gene density was expressed as the number of CpG sites and number of genes per 100 bp of nucleotide sequence respectively. The medium values were provided above each boxplot.
