# Supplementary material for: Hierarchical Clustering of Breast Cancer Methylomes Revealed Differentially Methylated and Expressed Breast Cancer Genes
Source: PLoS One. 2015 Feb 23;10(2):e0118453. doi: 10.1371/journal.pone.0118453 (PMC4338251; doi:10.1371/journal.pone.0118453)
Supplement: S12 Fig — The Restriction enzyme McrBC will specifically cleaving methylated CpG sites, leaving unmethylated DNA intact. The FISH signals were found at the nuclear periphery with chromosomal DNA counterstained with DAPI. (DOCX) [file pone.0118453.s012.docx]

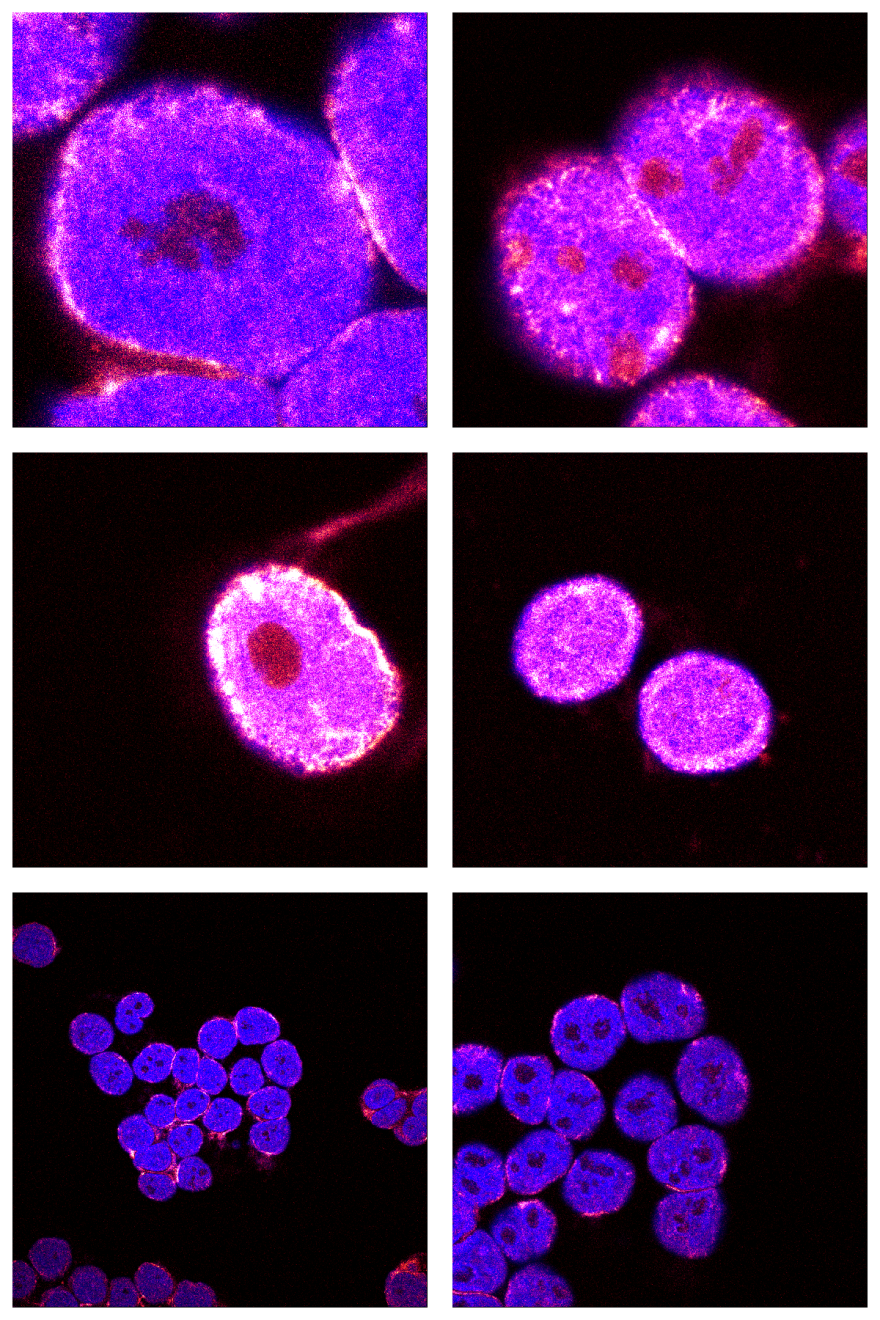


**Figure S12. Fluorescence *in situ* hybridization (FISH) analysis revealed localization of McrBC-resistant hypomethylated DNA in the MCF7 nuclei.** The Restriction enzyme McrBC will specifically cleaving methylated CpG sites, leaving unmethylated DNA intact. The FISH signals were found at the nuclear periphery with chromosomal DNA counterstained with DAPI.
