# Supplementary material for: Hierarchical Clustering of Breast Cancer Methylomes Revealed Differentially Methylated and Expressed Breast Cancer Genes
Source: PLoS One. 2015 Feb 23;10(2):e0118453. doi: 10.1371/journal.pone.0118453 (PMC4338251; doi:10.1371/journal.pone.0118453)
Supplement: S13 Fig — (A) The proportion of 127 fragile sites overlapped predicted PMDs of the seven methylomes. More than 50% of the fragile sites in BT198 and breast cell lines were associated with PMDs. nPMD: sites that do not overlapped PMD. PMD: sites that contain PMDs. (B) The distribution of fragile sites DNA methylation. The methylation levels of PMD-containing fragile sites were significantly lower than those not having PMDs in BT126, BT198, HMEC, HCC1954 and MCF7 (t-test, p-value < 0.01). (DOCX) [file pone.0118453.s013.docx]

**Figure S13. Common fragile sites are associated with hypomethylated breast tumor PMDs.** (A) The proportion of 127 fragile sites overlapped predicted PMDs of the seven methylomes. More than 50% of the fragile sites in BT198 and breast cell lines were associated with PMDs. nPMD: sites that do not overlapped PMD. PMD: sites that contain PMDs. (B) The distribution of fragile sites DNA methylation. The methylation levels of PMD-containing fragile sites were significantly lower than those not having PMDs in BT126, BT198, HMEC, HCC1954 and MCF7 (t-test, p-value < 0.01).
